# Effects of copper, zinc, and manganese source and inclusion during late gestation on beef cow–calf performance, mineral transfer, and metabolism

**DOI:** 10.1093/tas/txad097

**Published:** 2023-08-16

**Authors:** Emma L Stephenson, Abigail R Rathert-Williams, Ann L Kenny, Dusty W Nagy, Brian M Shoemake, Thomas B McFadden, Heather A Tucker, Allison M Meyer

**Affiliations:** Division of Animal Sciences, University of Missouri, Columbia, MO 65211, USA; Division of Animal Sciences, University of Missouri, Columbia, MO 65211, USA; Division of Animal Sciences, University of Missouri, Columbia, MO 65211, USA; School of Veterinary Medicine & Biomedical Sciences, Texas A&M University, College Station, TX 77843, USA; School of Veterinary Medicine & Biomedical Sciences, Texas A&M University, College Station, TX 77843, USA; Division of Animal Sciences, University of Missouri, Columbia, MO 65211, USA; Novus International, Inc., St. Charles, MO 63304, USA; Division of Animal Sciences, University of Missouri, Columbia, MO 65211, USA

**Keywords:** colostrum, developmental programming, milk, neonates, pregnancy, trace minerals

## Abstract

To determine effects of Cu, Zn, and Mn source and inclusion during late gestation, multiparous beef cows [*n* = 48; 649 ± 80 kg body weight (BW); 5.3 ± 0.5 body condition score (BCS)] were individually-fed hay and supplement to meet or exceed all nutrient recommendations except Cu, Zn, and Mn. From 91.2 ± 6.2 d pre-calving to 11.0 ± 3.2 d post-calving, cows received: no additional Cu, Zn, or Mn (control, CON), sulfate-based Cu, Zn, and Mn (inorganic, ITM) or metal methionine hydroxy analogue chelates (MMHAC) of Cu, Zn, and Mn at 133% recommendations, or a combination of inorganic and chelated Cu, Zn, and Mn (reduce and replace, RR) to meet 100% of recommendations. Data were analyzed with treatment and breeding group (and calf sex if *P* < 0.25 for offspring measures) as fixed effects, animal as experimental unit, and sampling time as a repeated effect for serum, plasma, and milk measures over time. Post-calving cow liver Cu was greater (*P *≤ 0.07) in MMHAC compared with all other treatments. Calves born to RR had greater (*P *≤ 0.05) liver Cu than ITM and CON, and MMHAC had greater (*P *= 0.06) liver Cu than CON. Liver Mn was less (*P *≤ 0.08) for RR calves than all other treatments. Calf plasma Zn was maintained (*P *≥ 0.15) from 0 to 48 h of age in ITM and MMHAC but decreased (*P *≤ 0.03) in CON and RR. Gestational cow BW, BCS, and metabolites were not affected (*P *≥ 0.13) by treatment, but gestational serum thiobarbituric acid reactive substances (TBARS) were greater (*P *= 0.01) for CON than MMHAC. Treatment did not affect (*P *≥ 0.13) calf birth size, vigor, placental size and minerals, or transfer of passive immunity. Neonatal calf serum Ca was greater (*P *≤ 0.05) for MMHAC than all other treatments; other calf serum chemistry and plasma cortisol were not affected (*P *≥ 0.12). Pre-suckling colostrum yield, and lactose concentration and content, were greater (*P *≤ 0.06) for MMHAC compared with ITM and RR. Colostral triglyceride and protein concentrations were greater (*P *≤ 0.08) for RR than MMHAC and CON. Cow lactational BW and BCS, milk yield and composition, and pre-weaning calf BW and metabolism were not affected (*P *≥ 0.13) by treatment. Lactational serum TBARS were greater (*P *= 0.04) for RR than CON at day 35 and greater (*P *≤ 0.09) for MMHAC at day 60 than all other treatments. Source and inclusion of Cu, Zn, and Mn altered maternal and neonatal calf mineral status, but calf size and vigor at birth, passive transfer, and pre-weaning growth were not affected in this study.

## Introduction

Nutritional demands of the cow increase during the last third of gestation due to exponential fetal growth and during lactation due to demands for milk production (NASEM, 2016). Both of these physiological states disrupt trace mineral homeostasis of the dam to partition trace minerals from the diet or storage (primarily liver) to the placenta (Mills and Davies, 1979) or the mammary gland (Annenkov, 1982). Because essential trace minerals Cu, Zn, and Mn are constituents of metalloenzymes and cofactors in many enzyme systems (McDowell, 1992), they are important in almost all normal biochemical processes (Spears, 1999). In reproducing females, these trace minerals are particularly important for the antioxidant defense system (McDowell, 1992; Spears and Weiss, 2008), which works to combat greater oxidative stress resulting from increased metabolic demand during late gestation and lactation (Sordillo, 2005). Pregnant and lactating females are also completely responsible for providing Cu, Zn, and Mn to the fetus and early postnatal calf (Hidiroglou and Knipfel, 1981); these are necessary for immune, endocrine, reproductive, skeletal, and nervous system development and function (Hostetler et al., 2003).

Beef cows are typically fed forage-based diets that are highly variable in trace minerals and their antagonists, which can result in mineral deficiency (McDowell, 1992). Therefore, it is recommended that beef cows are provided trace mineral supplements that can vary in source, including organic sources of Cu, Zn, and Mn (chelated with amino acids or amino acid analogues) that are potentially more bioavailable than inorganic salts (sulfates and oxides; Spears, 2003). Dam nutrition greatly impacts the uterine environment and can have long-term effects on the offspring by altering fetal development (Fowden et al., 2006; Wu et al., 2006). Although recent research has demonstrated that trace mineral source and inclusion during pregnancy alone may impact calf pre-weaning performance (Marques et al., 2016), this study did not include a maternal diet deficient in trace minerals and only considered one form of organic Cu, Zn, and Mn. We hypothesized that 1) providing chelated Cu, Zn, and Mn to late gestational beef cows would improve cow mineral status, which would improve calf mineral status and fetal development, and 2) the basal diet without Cu, Zn, and Mn supplementation would negatively impact calf mineral status and fetal development. Our specific objectives were to determine the effects of Cu, Zn, and Mn source and inclusion during late gestation on 1) beef cow gestational performance, mineral status, oxidative stress, fetal and placental growth, and colostrogenesis, 2) calf vigor at birth, transfer of passive immunity, neonatal and pre-weaning metabolism, and pre-weaning growth, and 3) carry-over effects on beef cow lactational performance and oxidative stress.

## Materials and Methods

All animal procedures were approved by the University of Missouri Animal Care and Use Committee (Protocol #9045) and took place at the University of Missouri Beef Teaching and Research Farm (Columbia, MO).

### Animal Management and Diets

#### Treatment diets and housing.

From 91.2 ± 6.2 d pre-calving to 11.0 ± 3.2 d post-calving, 48 multiparous, fall-calving Simmental-Angus crossbred beef cows were fed a basal diet of tall fescue-based hay supplemented to meet or exceed all nutrient recommendations (NASEM, 2016) except Cu, Zn, and Mn and were assigned to receive 1 of 4 treatments. These treatments were 1) basal diet with no additional Cu, Zn, or Mn (control; **CON**); 2) basal diet with Cu, Zn, and Mn sulfates to supply 133% of NASEM (2016) recommendations (inorganic trace minerals; **ITM**); 3) basal diet with metal methionine hydroxy analogue chelates (**MMHAC**) of Cu, Zn, and Mn (MINTREX chelated trace minerals, Novus International, Inc., St. Charles, MO) to supply 133% of NASEM recommendations (MMHAC); or 4) basal diet with Cu, Zn, and Mn sulfates and chelates (same sources as ITM and MMHAC) blended to supply 100% NASEM recommendations (reduce and replace strategy; **RR**). Cows were allocated by body weight [**BW**; initial BW = 649 ± 80 (SD throughout methodology) kg], body condition score (**BCS**; initial BCS = 5.3 ± 0.5), age (3 to 7 yr at calving; average = 4.3 ± 1.2 yr), and breeding group [artificial insemination (**AI**) or natural service] to treatment. Cows bred by AI (*n* = 36) underwent a standard estrus synchronization protocol and were bred to a single Angus sire with an expected calving date of September 17, 2017. Cows bred by natural service (*n* = 12) were exposed to 1 of 4 Angus-based bulls after AI and were selected based on estimated conception 20 to 30 d after AI at pregnancy diagnosis. Each breeding group began treatment diets at day 192 of gestation (estimated for natural service group).

Treatment diets were individually-fed using a Calan gate feeding system (Calan Broadbent Feeding System, American Calan, Northwood, NH). Cows were housed in partially-covered 3.7 × 15.8 m pens (*n* = 4 cows/pen, all treatments represented in each pen) with concrete floors bedded with sawdust. Each pen had 4 electronic feeding gates and an automatic waterer. Cows were acclimated to the electronic gate feeding system for ≥ 15 d prior to treatment initiation.

Three different tall fescue-based hay lots (Table 1) were fed during the treatment period, with all treatments receiving the same hay on any given calendar date. All hays were harvested and baled in late spring or early summer from the University of Missouri Beef Teaching and Research Farm, stored under roof, and then chopped prior to feeding in Calan gates. Treatments were delivered in a soyhull-based pelleted supplement that were formulated for each treatment based on initial core samples of the corresponding hay to meet or exceed net energy of maintenance (**NE**_**m**_), crude protein (**CP**), Ca, P, Na, I, Se, Co, vitamin A, vitamin D, and vitamin E recommendations (NASEM, 2016). Supplement inclusion of Cu, Zn, and Mn was formulated using initial hay core sample Cu, Zn, and Mn concentrations and individual treatment targets. Sulfate-based Cu, Zn, and Mn included in ITM and RR supplements were Cu sulfate pentahydrate, Zn sulfate monohydrate, and Mn sulfate monohydrate. Methionine hydroxy analogue supplement (MFP feed supplement, Novus International, Inc.) was included in CON, ITM, and RR supplements to provide similar amounts of methionine hydroxy analogue to that provided by the chelated Cu, Zn, and Mn source (MINTREX chelated trace minerals, Novus International, Inc.) fed to the MMHAC treatment cows.

**Table 1.** Nutrient composition and feeding duration of hays fed during the treatment period

Variable	Hay 1	Hay 2	Hay 3^1^
Days fed to AI breeding group^2^	37	32	18 to 53
Days fed to NS breeding group^2^	16	32	42 to 51
Dry matter, %	87.0	82.1	86.9
	---Dry matter basis---		
Crude protein, %	11.9	14.6	10.6
Neutral detergent fiber, %	67.2	57.1	62.4
Acid detergent fiber, %	43.7	43.9	40.9
Net energy of maintenance^3^, Mcal/kg	0.95	0.94	1.04
Cu, mg/kg	3.59	7.43	6.63
Zn, mg/kg	6.05	14.57	7.90
Mn, mg/kg	46.0	32.7	73.4

^1^Hay 3 feeding duration was dependent on calving date of individual cows; therefore, a range is given.

^2^Breeding groups: AI, cows bred by artificial insemination; NS, cows bred by natural service.

^3^Calculated value: 2.392 − 0.033 × (% acid detergent fiber).

All treatment supplements corresponding with hays 1 and 3 were composed of soyhulls, dried distillers’ grains with solubles (**DDGS**), choice white grease, calcium carbonate, salt, vitamin ADE (ADE NutraMix, Nutra Blend, LLC, Neosho, MO), calcium iodate, sodium selenite, and cobalt sulfate. Treatment supplements corresponding with hay 2 had similar composition but did not include DDGS due to hay CP concentration. Nutrient composition of treatment supplements corresponding with hays 1, 2, and 3 were 21.0%, 8.5%, and 25.1% CP [dry matter (**DM**) basis], respectively, and 1.74, 1.16, and 1.93 Mcal NE_m_/kg [DM basis; calculated based upon ingredient inclusion using NASEM (2016) values], respectively.

Supplement was fed every morning (0730 h) in feed pans in the Calan gates to prevent waste. Approximately equal amounts of hay were individually weighed and fed every morning (0800 h) and evening (1800 h). The amount of hay offered to each cow was initially calculated assuming 1.2% BW in neutral detergent fiber (**NDF**) intake of hay, and then adjusted based on actual intakes for each cow to have approximately 10% refusal rate. Hay refusals were weighed back weekly and sampled for DM analysis. Supplement was fed to be 11.5% of total dry matter intake (**DMI**) to meet treatment targets for Cu, Zn, and Mn, and was adjusted weekly based on hay disappearance the previous week. On the rare occasion there were supplement refusals, the refusal was fed to the same cow with the next day’s supplement.

To prevent calf health issues, cows were moved to 18 × 61 m drylot calving pens (described by Duncan and Meyer, 2019) by treatment at 17.3 ± 7.0 d pre-calving. In the calving pens, ad libitum hay (hay 3 in Table 1) was provided as round bales, treatment supplements were pen-fed at 1800 h, and cows had free access to water. The amount of supplement fed to each pen was calculated by estimating hay DMI using total pen BW and assuming cows would consume 1.2% BW of NDF. Supplement was fed to be 11.5% of total pen DMI, similar to when cows were individually-fed.

#### Nutrient analysis and intake calculations.

Daily grab samples of hay and supplement were collected and composited at 2-wk intervals, and hay core samples were collected from each bale upon delivery to the calving pens. All feed and refusal samples were first dried in a 55°C oven for a minimum of 48 h. Feed samples were ground and analyzed for NDF, acid detergent fiber, CP, and ash as described by Niederecker et al. (2018).

Average weekly DMI was calculated during individual feeding as hay DM refusals subtracted from hay DM offered, then added to supplement DM. After cows were moved to calving pens, hay DMI was estimated as 1.2% BW in hay NDF, and supplement DMI was estimated for each cow using the same equations used to calculate amount of supplement to feed each pen. Daily hay and supplement DMI were then multiplied by nutrient densities of corresponding subsamples to determine daily DM, CP, NE_m_, Cu, Zn, and Mn provided.

Dry matter intakes, dietary trace mineral densities and intakes, and target dietary trace mineral densities for each treatment are presented in Table 2. Actual dietary trace minerals provided varied from targets for ITM, MMHAC, and RR because composition of hay fed differed from the initial hay core samples used to formulate treatment supplements. This was likely due to high variability of trace minerals in forages (McDowell, 1992).

**Table 2.** Dry matter intakes and dietary trace minerals provided during the treatment period

Variable	Treatment^1^			
	CON	ITM	MMHAC	RR
Dry matter intake, kg/d				
Days 0 to 28 of study	10.7	11.4	10.9	10.9
Days 29 to 56 of study	12.2	12.7	12.2	12.2
Days 57 to 74 of study	12.3	13.0	12.2	12.5
Calving pens^2^	12.9	13.5	12.9	13.1
Dietary Cu, mg/kg dry matter (mg/d)				
*Target*^3^	—	*13.0*	*13.0*	*10.0*
Days 0 to 28 of study	4.5 (48)	12.0 (137)	15.2 (167)	9.7 (106)
Days 29 to 56 of study	6.7 (82)	13.7 (175)	15.9 (194)	11.1 (136)
Days 57 to 74 of study	7.0 (87)	14.7 (191)	16.7 (205)	12.4 (155)
Calving pens^2^	8.8 (114)	17.7 (238)	19.6 (253)	15.5 (204)
Dietary Zn, mg/kg dry matter (mg/d)				
*Target*^3^	—	*40.0*	*40.0*	*30.0*
Days 0 to 28 of study	9.8 (105)	30.5 (346)	32.1 (351)	23.0 (250)
Days 29 to 56 of study	15.2 (186)	37.0 (471)	39.4 (482)	29.6 (362)
Days 57 to 74 of study	16.9 (209)	40.5 (526)	41.7 (511)	33.7 (420)
Calving pens^2^	13.8 (178)	36.8 (495)	39.6 (512)	31.7 (417)
Dietary Mn, mg/kg dry matter (mg/d)				
*Target*^3^	*—*	*53.3*	*53.3*	*40.0*
Days 0 to 28 of study	43.2 (466)	60.3 (684)	64.7 (706)	51.3 (557)
Days 29 to 56 of study	36.2 (441)	55.5 (705)	61.7 (753)	45.0 (549)
Days 57 to 74 of study	47.4 (574)	61.1 (788)	65.6 (800)	56.8 (707)
Calving pens^2^	65.9 (849)	65.5 (880)	64.1 (827)	64.8 (853)

^1^Cows were individually -ed hay and supplemented with: no additional Cu, Zn, or Mn (control, CON), sulfate-based Cu, Zn, and Mn (inorganic, ITM) or methionine hydroxy analogue chelates of Cu, Zn, and Mn (MMHAC) to meet 133% of recommendations, or a combination of inorganic and chelated Cu, Zn, and Mn (reduce and replace, RR) to meet 100% of recommendations from 91.2 ± 6.2 d pre-calving until 11.0 ± 3.2 d post-calving.

^2^Cows were housed in drylot calving pens by treatment starting day 74 of study (average) until 11.0 ± 3.2 d post-calving, offered ad libitum hay, and pen-fed supplement. Intakes were calculated using estimated hay dry matter (1.2% BW NDF intake).

^3^Target concentrations of diets based on NASEM (2016) gestating cow Cu, Zn, and Mn recommendations and treatment targets.

Target should be italicized to indicate that those are targets, rather than the actual intakes.

#### Post-treatment management.

After treatment termination (11.0 ± 3.2 d post-calving), all cow–calf pairs were housed in an additional calving pen for ≥ 1 wk for monitoring, provided ad libitum tall fescue-based hay (hay 3; Table 1), and allowed access to a pressed, molasses-based supplement that provided vitamins and inorganic mineral sources (6.0 to 6.5% Ca, 3.0 to 3.5% NaCl, ≥ 6.0% P, ≥ 3.0% Mg, ≥ 3.0% K, ≥ 1,100 mg/kg Cu, ≥ 50 mg/kg I, ≥ 600 mg/kg Mn, ≥ 9 mg/kg Se, ≥ 1,600 mg/kg Zn, ≥ 41,000 IU/kg vitamin A, ≥ 99,225 IU/kg vitamin D3, and ≥ 287 IU/kg vitamin E; MLS #12 Minera-lix, Midcontinent Livestock Supplements, Moberly, MO). Cow–calf pairs were then moved to and rotated through 3 tall fescue-based pastures as a single group. They were fed harvested forage and/or grain supplement when pasture was limiting, provided ad libitum access to water and the same vitamin and mineral source, and monitored until weaning at 195.3 ± 8.3 d post-calving.

### Gestational Data Collection and Sampling

Two-day BW was collected before morning feeding prior to study initiation (“initial;” 2 d pre-study for AI and 9 d pre-study for natural service) and at days 28 and 56 of the treatment period. Single-day BW was collected in late gestation prior to moving to calving pens (“pre-calving;” 17.3 ± 7.0 d pre-calving, days 73.8 ± 3.1 of treatment). At study initiation and on days 28 and 56 of the study, 3 trained technicians recorded BCS (9-point scale: 1 = emaciated, 9 = obese; Wagner et al., 1988), which were then averaged for each cow at each timepoint. At initiation, day 28, day 56, and pre-calving, blood samples were collected into 4 tubes, including 2 Vacutainer serum collection tubes containing no additives (10 mL draw; Becton Dickinson, Franklin Lakes, NJ), 1 Monoject plasma collection tube containing 0.10 mL of 15% K_3_EDTA (10 mL draw; Covidien, Mansfield, MA), and 1 Vacutainer plasma collection tube containing 15 mg of sodium fluoride and 12 mg of potassium oxalate (6 mL draw; Becton Dickinson) for glucose determination. Blood tubes were inverted, placed on ice (serum tubes were allowed to clot before placing on ice), and centrifuged for 30 min at 1,500 × *g* at 4°C within 10 h of collection. Serum or plasma was pipetted in 2-mL microcentrifuge tubes at stored at −20°C until later analyses.

Initial liver biopsies were performed prior to study initiation (2 d pre-study for AI and 9 d pre-study for natural service). To prepare the biopsy site, organic matter was removed from the surgical area, hair was clipped, and the area was scrubbed with iodopovidone followed by 70% alcohol ≥ 3 times. The skin incision was made with a #10 scalpel blade, then the biopsy was taken using a stainless-steel trocar based on the procedure of Davies and Jebbett (1981), except that suction was not generally used during biopsy. The incision was closed with a single chromic gut suture, and the surrounding area was sprayed with 5% permethrin (Prozap Screw Worm Aerosol, Neogen Corporation, Pleasantville, IA). Samples were placed in a sterile plastic 2-mL microcentrifuge tube, flash frozen on dry ice, and then stored at −80°C for mineral analysis at a later date.

### Peripartum and Neonatal Data Collection and Sampling

#### Calf vigor and size at birth.

In the calving pens, cows were closely monitored by trained personnel to detect stage II parturition as described in Wichman et al. (2022) by walking through pens at least once every hour except between 0200 and 0400 h during heavy calving. Once stage II was detected, each cow was continuously monitored to record time of birth (complete expulsion of calf). If more than 1 h had passed since fetal membranes were observed or the calf was presenting abnormally, the cow was moved to the chute and assistance was provided (2 CON, 2 MMHAC, and 1 RR).

After birth, each calf was monitored to record time of standing (defined as calf being up on all 4 feet for 5 consecutive seconds) for calculation of time to stand, and at 10 min of age each calf was assigned a vigor score (1 = very weak to 5 = extremely vigorous) as described by Duncan et al. (2022). Calf birth weight, shoulder to rump length, heart girth, abdominal girth, flank girth, and cannon circumference were determined at 1.36 ± 2.70 h of age as described by Redifer et al. (2023). Calves were also identified with an ear tag, and the navel was sprayed with chlorhexidine solution until saturated. One calf (CON) was given colostrum composited from its dam’s treatment (composited after subsampling) due to high blood content in its dam’s colostrum.

#### Maternal blood, colostrum, and neonatal blood collection.

After each calf stood but before it suckled, both cow and calf were removed from the pen, and the cow was directed into a nearby chute. Cow jugular blood samples were collected (76.3 ± 77.5 min post-calving) into the same 4 tubes and processed as previously described for gestational blood sampling. The most full rear quarter was selected based upon visual inspection and palpation and was hand-milked completely prior to the calf suckling (57.5 ± 17.5 min post-calving) at described in Rathert-Williams et al. (2023). Colostrum volume and weight were recorded, and subsamples were aliquoted and stored at −20°C for later analysis.

Calf jugular blood samples were collected at 0 h of age (post-standing but pre-suckling; 34.3 ± 20.2 min of age), and at 48 h of age (48.2 ± 0.5 h of age). Blood was collected into 4 tubes at each time point, including 2 Vacutainer serum collection tubes containing no additives, 1 Monoject plasma collection tube containing 0.10 mL of 15% K_3_EDTA, and 1 Vacutainer plasma collection containing 10.8 mg of K_2_EDTA (6 mL draw; Becton Dickinson, Franklin Lakes, NJ) for mineral determination, and processed as previously described for cow samples.

#### Placenta collection and processing.

Cows were monitored closely post-calving, and placentas were collected after expulsion. Placentas were then rinsed and dissected as described by Redifer et al. (2021). During dissection, 3 large cotyledons were selected to be frozen for Cu, Zn, and Mn analysis. Wet weights of the selected cotyledons were recorded, and they were stored at −20°C until further analysis. After all placentas were collected and processed, DM weight was determined for cotyledonary and intercotyledonary tissues (except for the cotyledons removed for mineral analysis). The mineral analysis subsample was included in cotyledonary dry weight by adding wet weight of the subsample multiplied by the percent DM of cotyledonary tissue for that animal.

#### Maternal and neonatal liver biopsies.

Liver biopsies were performed on cows at 11.0 ± 3.2 d post-calving as described for initial cow liver sampling. Neonatal calf liver biopsies were conducted at 11.0 ± 3.2 d of age to prevent hyperthermia caused by the sedative xylazine (EMEA, 1999). Because biopsies may have occurred on days with high temperature humidity index, the target biopsy age range was 7 to 14 d of age to decrease heat stress to neonatal calves. Calves received 30 mg/kg BW oxytetracycline and 2.0 mg/kg BW flunixin meglumine (Hexasol, Norbrook Inc., Overland Park, KS) subcutaneously prior to biopsy as prophylaxis. The biopsy protocol for calves was based on Swanson et al. (2000). Calves were sedated with 0.05 to 0.12 mg/kg BW xylazine HCl (depending on calf size and vigor; AnaSed, Akorn Animal Health, Lake Forest, IL) via intravenous injection. After each calf was successfully sedated and placed in a left laterally recumbent position, the biopsy site was prepared similarly to cows. One milliliter of 2% lidocaine HCl (MWI Veterinary Supply, Boise, ID) was injected subcutaneously and intramuscularly at the surgical site, and an approximately 1-cm skin incision was made in the intercostal space between the 11th and 12th ribs on their right side approximately 15 cm from dorsal midline. A small trocar and cannula (bone marrow biopsy/aspiration needle; Jamshidi 8-gauge, 10 cm tapered distal tip, Becton Dickinson, New Franklin, NJ) was inserted through intercostal muscles and then into the peritoneal space. The stylet was removed, and the cannula was advanced into the liver with a twisting motion while angling the cannula towards the left elbow joint. The cannula was retracted, and the liver samples were placed in sterile plastic 2-mL microcentrifuge tubes. Samples were flash frozen on dry ice and stored at −80°C for later analysis. After biopsy, the incision site was treated as described for cows. Sedation was reversed using tolazoline (Tolazoline100 mg/mL, Akorn Animal Health) injected intravenously, which was dosed by administering the same volume as xylazine, resulting in a tolazoline dose of 0.26 to 0.59 mg/kg BW. Cow–calf pairs were monitored in a shaded area until the calf was fully recovered from sedation, and then they were moved to an extra calving pen for postbiopsy monitoring, and treatments were terminated.

### Lactation and Pre-weaning Data Collection

#### Cow performance and milk yield.

Single-day cow BW, BCS (by 2 trained technicians), and milk yields were collected at day 35 (34.6 ± 1.8 d post-calving) and day 60 (60.7 ± 3.7 d post-calving) of lactation. Milk yields over 4 h were measured in the evening (average: 2002 ± 1.2 h, range: 1734 to 2303 h) as described by Rathert-Williams et al. (2023). Briefly, cows were administered oxytocin, machine-milked, and then each quarter was hand-stripped to ensure complete removal of milk. Calves were separated from dams and housed in pens without fence line contact with cows, and cows were provided free access to water. The same milking protocol was performed 4.0 ± 0.1 h after initial milking to determine 4-h milk yields, which were then multiplied by 6 to estimate 24-h milk production. Milk weight and volume for each cow were recorded only at the second collection, and subsamples were aliquoted and stored at −20°C until composition analysis. Cows with 1 nonfunctional teat were included in the data set because their yields were not outliers. Prior to oxytocin administration at the initial milking, jugular blood samples were collected and processed as described for gestational blood sampling.

Cow 2-d BW was collected and BCS were recorded by 2 trained technicians when calves were weaned at 195.3 ± 8.3 d post-calving (single date for all calves).

#### Pre-weaning calf data collection and sampling.

Calf single-day BW was recorded at days 35, 60, and 125 of age (34.6 ± 1.8 d, 60.5 ± 4.3 d, and 125.9 ± 3.6 d of age, respectively) and 2-d BW was recorded immediately prior to weaning (195.3 ± 8.3 d of age). Jugular blood samples were also collected during these sampling times into the same 4 tubes as cow sampling. Blood samples were processed as previously described for cows.

### Lab Analyses

#### Tissue and feed mineral analysis.

Hay, supplement, colostrum and milk, neonatal calf plasma, and cotyledon Cu, Zn, and Mn analyses were performed by Novus International, Inc. using inductively coupled plasma (**ICP**) optical emission spectrometry with a cyclonic spray chamber and high solids GemCone nebulizer (PerkinElmer, Waltham, MA). Liver Cu, Zn, and Mn were determined by Novus International, Inc. using ICP mass spectrometry (Agilent 7500, Agilent Technologies, Santa Clara, CA). Samples were digested using HNO_3_ acid and heat (260°C). After acid digestion, an internal standard (0.2 mL of 250 ppm yttrium solution) was added, and all samples were brought up to a common volume using deionized water and mixed thoroughly. After cooling to room temperature, samples were centrifuged at 3,650 rpm for 15 min and filtered through a 0.2 µm nylon filter. A calibration curve was generated for each mineral. Samples were injected, normalized to the internal standard, and analyzed in triplicate. Analysis was considered acceptable if the tested value and theoretical value of the internal standard had a relative percent difference < 10%. Neonatal calf plasma Mn concentrations were not detectable (0.05 ppm detection limit) in any sample. Total cotyledonary mineral content was calculated by multiplying mineral concentration (DM basis) by total cotyledonary DM weight, and total colostrum or milk mineral content were calculated by multiplying mineral concentration by yield (weight).

#### Calf liver metallothionein mRNA expression.

Total messenger ribonucleic acid (**mRNA**) was extracted from 5 to 10 mg of calf liver using the RNeasy Mini Kit (Qiagen, Hilden, Germany) following manufacturer instructions. Ribonucleic acid (**RNA**) quantity of each sample was measured using a NanoDrop 1000 Spectrophotometer (Thermo Fisher Scientific), and RNA quality was assessed using gel electrophoresis. Extracted RNA was kept at −80°C until complementary DNA (**cDNA**) synthesis was performed using the QuantiTect Reverse Transcription kit (Qiagen) following manufacturer instructions. The resulting cDNA was diluted 1:5 in DNase/RNase-free water, and subsamples were pooled for use as an internal control. Samples and control were stored at −20°C until mRNA expression analysis using real-time polymerase chain reaction (**RT-PCR**).

Real-time PCR amplification of bovine metallothionein-1A and ribosomal subunit 9 (**S9**; reference gene) for each sample was performed in duplicate using iTaq Universal SYBR Green Supermix (Bio-Rad Laboratories Inc., Hercules, CA). Conditions for the RT-PCR reactions were 95°C for 30 s, 40 cycles of 95°C for 5 s followed by 56°C for 30 s, then a melt curve analysis was performed by increasing the temperature from 65 to 95°C in 0.5°C increments for 5 s each. Primer sequences (listed 5ʹ to 3ʹ) were bovine metallothionein-1A forward, CTGCTCCTGCCCCAC [56.2°C melting temperature (***T*_m_**)], reverse, CAGCCCTGGGCACAC (56.9°C *T*_m_); S9 forward, GAAGCTGATCGGCGAGTATG (55.6°C *T*_m_), reverse, CGCAACAGGGCATTACCTTC (56.6°C *T*_m_; Richard, 2008). DNase/RNase-free water was used as a no template control for each gene to ensure there was no contamination. Pooled cDNA sample was used to make a 4-point standard curve (1:1 to 1:1,000 dilutions) for each gene to calculate primer efficiency. Efficiencies for metallothionein 1A and S9 primers were 99.1% and 102.1%, respectively. Intraassay CV for metallothionein 1A and S9 Ct were 0.8% and 1.0%, respectively. The cycle threshold for the pooled internal control at a 1:5 dilution was calculated by using the log transformed linear curve produced by the standard dilutions included for both metallothionein 1A and S9. Expression of metallothionein 1A was then calculated relative to the reference gene and the pooled sample control (at 1:5 dilution) using the 2^−ΔΔCt^ method (Livak and Schmittgen, 2001). For mRNA expression data, if a 2^−ΔΔCt^ was > 3 SD away from the mean, it was considered an outlier (1 RR) and removed from the data set.

#### Circulating metabolites, blood chemistry, and cortisol.

Gestational and 1 h post-calving cow plasma glucose, serum urea N, and serum non-esterified fatty acids (**NEFA**) were analyzed using commercially available kits as described by Niederecker et al. (2018). Additionally, neonatal calf (0 and 48 h) and pre-weaning calf serum NEFA, as well as pre-weaning calf plasma glucose and serum urea N, were analyzed using these methods. Neonatal calf plasma triglycerides were analyzed as described by Larson-Peine et al. (2022). For each assay, samples were analyzed in duplicate, and pooled samples were used as controls. The intraassay and interassay CV were < 3.8% and < 5.4%, respectively.

Neonatal calf serum chemistry analysis was conducted by the University of Missouri Veterinary Medical Diagnostic Laboratory (**VMDL**) as described by Larson-Peine et al. (2022). This included serum glucose, urea N, creatinine, total protein, globulin, albumin, sodium, calcium, chloride, phosphorus, potassium, magnesium, anion gap, bicarbonate, direct and total bilirubin, aspartate aminotransferase (**AST**), gamma-glutamyl transferase (**GGT**), and creatine kinase (**CK**). Neonatal calf plasma cortisol was analyzed using a commercial coated-tube radioimmunoassay kit (MP Biomedicals, Irvine, CA) in duplicate as described previously (Foote et al., 2016) with an intraassay CV of 1.7%.

#### Calf serum immunoglobulins.

Calf serum from the 48-h sampling was analyzed for immunoglobulins (**Ig**) G, A, and M using sandwich enzyme linked immunosorbent assay (**ELISA**) kits (Bovine IgG ELISA Quantitation Set, Bovine IgA ELISA Quantitation Set, and Bovine IgM ELISA Quantitation Set, Bethyl Laboratories, Inc., Montgomery, TX), per manufacturer’s instructions. Pooled serum was used as an internal control, and samples were plated in duplicate on 96-well polystyrene plates and read at 450 nm on a UV–visible microplate reader. Coefficients of variation < 15% were considered acceptable. The intraassay and interassay CV were 4.1% and 7.7% for IgG, 4.9% and 5.7% for IgA, and 4.5% and 8.2% for IgM, respectively.

#### Cow serum oxidative stress markers.

Gestational, 1 h post-calving, and lactational cow serum were analyzed by Novus International, Inc. for thiobarbituric acid reactive substrates (**TBARS**) concentration using a commercially-available kit (Cayman Chemical, Ann Arbor, MI), as well as glutathione peroxidase (**GPx**), reduced glutathione (**GSH**), and oxidized glutathione (**GSSG**) concentration using an ELISA assay (GSH-Px ELISA kit, GSH ELISA kit, and GSSG ELISA kit, MyBioSource, Inc., San Diego, CA). Samples were analyzed in duplicate using a microplate reader (Epoch 2, BioTek, Winooski, VA) at 532 nm for TBARS assay and 450 nm for GPx, GSH, and GSSG assays. Intraassay and interassay CV for TBARS, GPx, GSH, and GSSG were < 3.0% and < 2.7%, respectively. Serum protein concentrations were used to report GPx, GSH, and GSSG concentrations relative to serum protein to ensure these oxidative stress marker concentrations were not confounded by serum dilution. Serum was analyzed in duplicate for protein concentration using a commercially available Coomassie (Bradford) Protein Assay Kit (Thermo Scientific, Waltham, MA). Intraassay and interassay CV were 5.2% and 2.9%, respectively.

#### Colostrum and milk quality.

Colostrum and milk were analyzed for lactose, triglycerides (as a measure of fat), protein, and urea N as described in Rathert-Williams et al. (2023). All samples were analyzed in duplicate, and pooled colostrum and milk samples were used as internal controls. The intraassay and interassay CV were < 4.2% and < 8.4%, respectively. Colostral IgG, IgA, and IgM were also determined as described for calf serum. The intraassay and interassay CV for IgG were 3.0% and 2.4%, respectively, for IgA were 7.0% and 12.1%, respectively, and for IgM were 3.9% and 4.6% respectively. Total nutrient content of colostrum and milk and total Ig content of colostrum were calculated by multiplying the nutrient or Ig concentration by the volume of sample collected.

### Statistical Analysis

Four cows were removed from the study (3 late gestational abortion, 1 cow that was earlier in pregnancy than diagnosed), resulting in 44 cows that calved. After post-calving liver biopsies, 1 cow was removed due to temperament resulting in 43 cow–calf pairs that were followed through weaning. Neonatal calf data and sampling *n* varied due to impermissible dam temperament or unknown time of birth. Incomplete placentas or those consumed by cows were not included. Neonatal calf data were excluded for a twin (1 stillborn and 1 live calf), but that cow–calf pair was included in other pre-weaning data. One cow was not included in milk yields due to poor udder conformation but included for all other pre-weaning sampling. Final *n* are given in data tables and figures.

Data were analyzed using the MIXED procedure in SAS 9.4 (SAS Institute Inc., Cary, NC) with gestational treatment and breeding group as fixed effects and cow as experimental unit. For all offspring measures, calf sex was included in the model as a fixed effect if *P* < 0.25. Initial cow liver mineral concentration was used as a covariate for post-calving cow liver mineral concentration. Calf birth BW and age at biopsy were used as covariates for calf liver mineral concentrations and relative metallothionein 1A mRNA expression. Gestational treatment, sampling time, and their interaction were also considered fixed effects for cow or calf circulating metabolites, serum chemistry, and oxidative stress markers, as well as milk yield and composition over time. These were analyzed as repeated measures, using the best-fit covariate structure (chosen from compound symmetry, heterogeneous compound symmetry, autoregressive, and heterogenous autoregressive). Cow circulating metabolites at 1 h post-calving were considered independently of gestation or lactation. Main effects of sampling time will not be discussed. Means were separated if the treatment or treatment × time *P *≤ 0.10. Least square means were separated using least significant difference, with differences considered significant when *P* ≤ 0.05 and tendencies when 0.05 < *P* ≤ 0.10.

## Results and Discussion

### Copper, Zinc, and Manganese Status

#### Cow mineral intakes.

Intakes of Cu, Zn, and Mn (both total and density in diet; Table 2) were affected (*P* ≤ 0.002) by gestational treatment throughout the study, except for Mn intake while in the calving pens (*P* ≥ 0.25). Hay 3 had adequate Mn density, which prevented delivery of Mn targets. Mineral density of the diet varied from targets due to cow DMI and variation in hay mineral concentration. Intake of Cu from days 0 to 28 and days 28 to 56 of the study was less (*P *≤ 0.02) for ITM than MMHAC cows. Otherwise, treatment targets for CON to have less Cu, Zn, and Mn intake than other treatments and MMHAC and ITM to have similar Cu, Zn, and Mn intakes that were in excess of recommendations were accomplished (*P* < 0.11). Dry matter intake was not affected (*P* ≥ 0.59) by gestational treatment (Table 2).

#### Cow mineral status.

Initial cow liver Cu concentrations were not different (*P =* 0.44) among treatments (Table 3). Prior to treatment initiation, most cows were in marginal (33 to 125 mg/kg DM; 4 CON, 4 ITM, 4 MMHAC, and 5 RR) or adequate (125 to 600 mg/kg DM; 7 CON, 6 ITM, 4 MMHAC, 5 RR) liver Cu status based on Kincaid (2000). One cow was within clinically deficient (< 20 mg/kg DM; RR) and 3 cows were within deficient (< 33 mg/kg DM; 2 MMHAC and 1 RR) ranges pre-study (Kincaid, 2000).

**Table 3.** Effects of Cu, Zn, and Mn source and inclusion during late gestation on cow and calf liver Cu, Zn, and Mn status

Variable	Treatment^1^				SEM	*P*-value
	CON	ITM	MMHAC	RR		
Cow age, yr	3.99	4.38	4.02	4.17	0.44	0.88
Cow liver						
Cu, mg/kg dry matter						
Initial^2^	143	144	147	101	25	0.44
Post-calving^3^	100^b^	131^b^	214^a^	88^b^	33	0.03
Zn, mg/kg dry matter						
Initial^2^	141	146	123	115	16	0.41
Post-calving^3^	151	112	149	136	20	0.43
Mn, mg/kg dry matter						
Initial^2^	8.93	8.71	8.94	7.53	0.78	0.45
Post-calving^3^	9.70	7.94	9.83	8.45	0.90	0.28
Calf liver^4^						
Cu, mg/kg dry matter	174^c^	182^bc^	221^ab^	228^a^	18	0.05
Zn, mg/kg dry matter	191	166	148	179	24	0.58
Mn, mg/kg dry matter	8.55^a^	8.53^a^	8.36^a^	7.24^b^	0.47	0.09
Metallothionein 1A relative mRNA^5^ expression, 2^-ΔΔCt^	1.60	1.21	0.71	1.68	0.57	0.58

^1^Cows were individually-fed hay and supplemented with: no additional Cu, Zn, or Mn (control, CON), sulfate-based Cu, Zn, and Mn (inorganic, ITM) or methionine hydroxy analogue chelates of Cu, Zn, and Mn (MMHAC) to meet 133% of recommendations, or a combination of inorganic and chelated Cu, Zn, and Mn (reduce and replace, RR) to meet 100% of recommendations from 91.2 ± 6.2 d pre-calving until 11.0 ± 3.2 d post-calving.

^2^Initial = 94.2 ± 5.5 d pre-calving; prior to gestational treatment initiation. CON *n* = 11, ITM *n* = 11, MMHAC *n* = 10, RR *n* = 12.

^3^Post-calving = 11.0 ± 3.2 d post-calving. CON *n* = 11, ITM *n* = 11, MMHAC *n* = 9, RR *n* = 12.

^4^Liver biopsies collected at 11.0 ± 3.2 d of age. CON *n* = 11, ITM *n* = 10, MMHAC *n* = 10, RR *n* = 11.

^5^mRNA, messenger ribonucleic acid.

^abc^Within an item, treatment means differ (*P* < 0.10).

Gestational treatment affected (*P *= 0.03) post-calving cow liver Cu concentration, where cows fed MMHAC had greater (*P *≤ 0.01) liver Cu than cows fed CON and RR, and MMHAC tended (*P *= 0.07) to have greater liver Cu than ITM-fed cows. The liver is the main storage organ for Cu (McDowell, 1992), making liver Cu concentration the best indicator of its status (Herdt and Hoff, 2011). Our data suggest that the Cu-methionine hydroxy analog chelate was more bioavailable than Cu sulfate, as cows fed MMHAC tended to have greater liver Cu than ITM even though Cu inclusion was similar for both treatments. Post-calving, 1 cow was within clinically deficient (ITM), 3 cows were within deficient (2 CON, 1 MMHAC), and the rest were within marginal (5 CON, 3 ITM, 1 MMHAC, 8 RR) and adequate (4 CON, 7 ITM, 7 MMHAC, 3 RR) classifications based on Kincaid (2000). Cows fed 133% of Cu recommendations (NASEM, 2016) during late gestation were more likely to have adequate Cu status in the current study, which suggests that late gestational dietary Cu recommendations may be underestimated. Cows fed to meet NASEM (2016) Cu recommendations in the RR treatment did not improve Cu status, and their average liver Cu was within the marginal status (Kincaid, 2000) after > 100 d of this diet.

Initial cow liver Zn and Mn concentrations were not different (*P *≥ 0.41) among treatments (Table 3), and gestational treatment did not affect (*P *≥ 0.28) cow liver Zn or Mn concentrations post-calving. Cow liver Zn values were predominantly within the adequate Zn status (25 to 200 mg/kg DM) published by Kincaid (2000), with only 2 initial cows (1 MMHAC and 1 RR) and 2 post-calving cows (1 ITM and 1 RR) within the marginal Zn status and 1 post-calving cow (CON) within the high Zn status of Kincaid (2000). No liver Mn ranges have been shown to predict Mn status accurately due to the high variability of liver Mn (Spears et al., 2022). Liver Zn and Mn concentrations are less responsive to changes in dietary trace minerals than liver Cu (Kincaid, 2000; Herdt and Hoff, 2011) and are not reliable indicators of Zn and Mn status (Spears et al., 2022).

In a similar study, mature beef cows provided ad libitum access to inorganic or organic (proteinates) Cu, Zn, and Mn in late gestation and lactation had greater liver Cu, Zn, and Mn at day 110 of lactation than cows that did not receive supplement (Ahola et al., 2004). In the same study, cows fed the organic source of minerals had greater liver Cu than cows fed the inorganic source, indicating the organic source had greater bioavailability as average daily mineral disappearance was similar among treatments (Ahola et al., 2004). Marques et al. (2016) reported that beef cows supplemented with inorganic (sulfate) or organic sources (amino acid complexes) of Cu, Zn, and Mn in excess of recommendations during late gestation had greater pre-calving liver Cu and Zn compared with cows receiving no supplemental minerals, and liver Cu was greater for cows fed inorganic sources than organic. Feeding of the same organic sources above recommendations to beef cows during mid- and late gestation resulted in greater liver Cu and Zn in cows fed inorganic sources (Harvey et al., 2021a). In another study, Cu, Zn, and Mn supplementation in excess of recommendations from inorganic or organic (amino acid complexes) sources from calving until breeding resulted in 2-yr old cows with greater liver Cu, but similar liver Zn and Mn concentrations, compared with unsupplemented controls (Olson et al., 1999). Dairy cows provided daily boluses of organic sources of Cu, Zn, and Mn (amino acid complexes) during late gestation had greater liver Cu and Mn at day 10 post-calving compared with cows provided inorganic sources, with no difference between liver Zn concentrations (Osorio et al., 2016). Although duration of supplementation, source of trace minerals, breed type, and sampling times differed among these studies, these data demonstrate that liver Cu, Zn, and Mn can be affected by source of supplemental trace minerals.

#### Neonatal calf plasma minerals.

**Figure 1.**
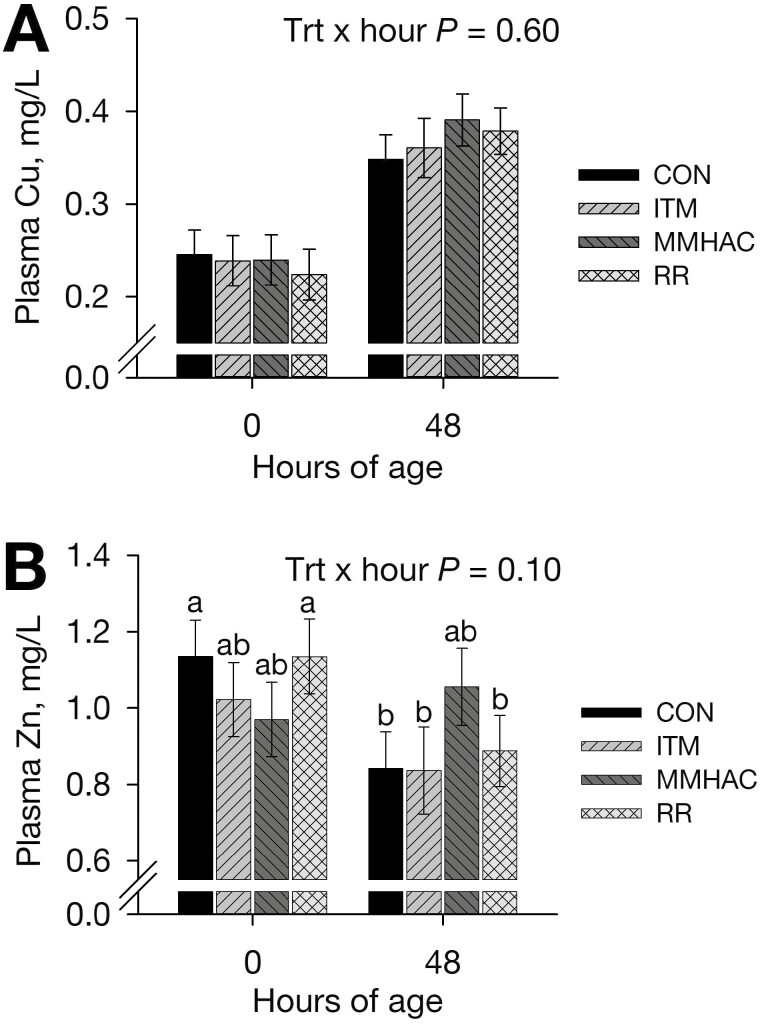
Effects of Cu, Zn, and Mn source and inclusion during late gestation on neonatal plasma Cu (Panel A) and Zn (Panel B) concentrations (plasma Mn was not detectable). Cows were individually-fed hay and supplemented with: no additional Cu, Zn, or Mn (control, CON), sulfate-based Cu, Zn, and Mn (inorganic, ITM) or methionine hydroxy analogue chelates of Cu, Zn, and Mn (MMHAC) to meet 133% of recommendations, or a combination of inorganic and chelated Cu, Zn, and Mn (reduce and replace, RR) to meet 100% of recommendations from 91.2 ± 6.2 d pre-calving until 11.0 ± 3.2 d post-calving. Samples at 0 h were collected post-standing but pre-suckling (34.3 ± 20.2 min of age). Least square means ± SEM are presented (CON *n* = 11, ITM *n* = 8 to 10, MMHAC *n* = 9 to 10, RR *n* = 10 to 11). ^a,b^Means differ (*P *≤ 0.10).

Neonatal plasma Cu concentration was not affected (*P *≥ 0.60) by gestational treatment or treatment × hour (Fig. 1A); however, calf plasma Cu increased (*P *< 0.001) from 0 to 48 h of age. This rapid increase is expected in neonatal ruminants (Spears et al., 2022) and was likely due to an increase in ceruloplasmin production that occurs in the first weeks of life and acts as the main Cu transporter in blood (Underwood and Suttle, 1999). In this study, only 2 calves at 48 h had plasma Cu above the 0.6 mg/L threshold put forth by Underwood (1981) for adults, highlighting the low plasma Cu observed in neonates. Herdt and Hoff (2011) published reference intervals of 0.3 to 1.0 mg/L for neonates, of which 6 calves in the current study were below at 48 h (all > 0.2 mg/L; 1 CON, 1 ITM, 1 MMHAC, 3 RR). Plasma or serum Cu is indicative of the Cu transport pool (Herdt and Hoff, 2011), which was not affected by late gestational trace mineral source or inclusion at birth or 48 h of age. Similar results have been reported previously, where plasma or serum Cu was not affected by maternal trace mineral supplementation (Muehlenbein et al., 2001; Jacometo et al., 2015; Stokes et al., 2018).

There tended (*P *= 0.10) to be a gestational treatment × sampling hour interaction for plasma Zn concentrations (Fig. 1B). Gestational treatment did not affect (*P *≥ 0.13) calf plasma Zn within sampling hour. Despite this, plasma Zn concentrations were maintained from 0 to 48 h of age in calves born to MMHAC (*P* = 0.47) and ITM (*P* = 0.15) cows, whereas plasma Zn concentrations decreased from 0 to 48 h in CON (*P* = 0.009) and RR (*P* = 0.03). At 0 h, 4 calves (2 ITM, 2 MMHAC) had plasma Zn below the reference interval (0.6 to 1.75 mg/L) for neonates proposed by Herdt and Hoff (2011), and 3 calves (1 CON, 1 ITM, and 1 RR) were below this range at 48 h. Circulating Zn is responsive to dietary changes, which makes it a good indicator of Zn status (Herdt and Hoff, 2011), and ruminants are born with greater circulating Zn than their dams (Spears et al., 2022). In this study, colostral Zn (discussed in later section) was not affected by gestational treatment, suggesting that maintenance of plasma Zn in calves born to dams fed 133% of Cu, Zn, and Mn recommendations was not a function of greater Zn intake. It is possible that calves born to MMHAC and ITM dams had greater liver Zn at birth, the mobilization of which allowed for greater circulating Zn, then resulted in similar liver Zn at day 11 of age (discussed in later section). Harvey et al. (2021a) reported that neonatal calf liver Cu, Zn, and Mn decreased during the first 24 h of life, suggesting that mineral mobilization from liver is likely during this period. Liver mineral concentrations at birth would give better insight into this hypothesis. These data also suggest that gestational Zn recommendations may not allow for optimal neonatal Zn status, as calves born to cows fed in excess of Zn recommendations (MMHAC and ITM) were able to maintain plasma Zn, unlike those born to cows fed a marginally Zn-deficient diet (CON) or fed to meet Cu, Zn, and Mn recommendations (RR). Providing supplemental Zn to cows (Muehlenbein et al., 2001) or heifers (Stokes et al., 2018) did not alter neonatal calf plasma or serum Zn in previous studies. However, calf plasma Zn decreased from birth to 1 d of age for those born to cows with daily organic trace mineral boluses 30 d prepartum, whereas calves born to cows that received inorganic trace mineral boluses had no change (Jacometo et al., 2015). As the calves had similar mineral intakes from colostrum, Jacometo et al. (2015) hypothesized that maternal trace mineral intake in excess of recommendations altered Zn metabolism.

Neonatal calf plasma Mn did not reach the detection limit (0.05 mg/L) in this study. Plasma Mn is generally low, and not indicative of Mn status (Spears et al., 2022).

#### Calf liver mineral and metallothionein.

Gestational treatment affected (*P *= 0.05) calf liver Cu concentration at day 11 of age, where calves born to cows fed RR had greater (*P *≤ 0.05) liver Cu compared with ITM and CON calves, and MMHAC calves tended (*P *= 0.06) to have greater liver Cu compared with CON calves (Table 3). Four calves (2 CON, 1 ITM, 1 MMHAC) had liver Cu less than neonatal reference intervals (125 to 650 mg/kg DM) proposed by Herdt and Hoff (2011). Liver Cu stores are greater at birth than in older animals to make up for low Cu in milk and provide Cu for ceruloplasmin transport, and Cu transfer from dam to fetus is greater in late gestation (Spears et al., 2022). Results indicate calves born to cows fed RR and MMHAC had improved Cu status compared with those fed a marginally Cu-deficient diet during gestation (CON), which may be due to greater Cu transfer during late gestation. Greater liver Cu in RR and MMHAC, but not ITM, compared with CON suggests that source of maternal Cu may have been more important than inclusion in excess of recommendations. Greater liver Cu in MMHAC cows than all other treatments at the same sampling time could indicate gestational supplementation of both inorganic and chelated Cu, Zn, and Mn together (RR) altered perinatal Cu metabolism, benefiting neonatal Cu stores more than maternal Cu stores.

Gestational treatment did not affect (*P *= 0.58) calf liver Zn concentrations (Table 3). Fetal liver Zn is greater than maternal liver Zn (Spears et al., 2022), so similar cow and calf liver values observed here may indicate postnatal Zn mobilization. Using the Herdt and Hoff (2011) neonatal reference interval (120 to 400 mg/kg DM), 60% of calves were within this range (7 CON, 6 ITM, 5 MMHAC, 6 RR), with 1 above (CON) and the rest below (3 CON, 5 ITM, 4 MMHAC, and 5 RR). Post-calving cow liver Zn was not affected by treatment; therefore, similar calf liver Zn concentrations among treatments were anticipated. Although the liver stores Zn, circulating Zn may be a better representation of trace mineral status because it is more responsive to dietary changes and Zn is widely distributed throughout the body (Herdt and Hoff, 2011).

Calf liver Mn concentration tended (*P *= 0.09) to be affected by gestational treatment, where calves born to cows fed RR had less (*P *≤ 0.04) liver Mn compared with ITM and CON calves and tended (*P *= 0.08) to have less liver Mn than MMHAC calves (Table 3). These results were unexpected as cow liver Mn was not affected by gestational treatment, and the basal diet provided adequate Mn for the majority of the treatment period. Previously, beef heifers fed a Mn-deficient diet throughout gestation gave birth to neonatal calves with less liver Mn than heifers fed a Mn-adequate diet (Howes and Dyer, 1971). Despite this, liver Mn is poorly responsive to dietary changes and not necessarily indicative of Mn status (Spears et al., 2022).

Both calf Cu and Zn have previously been responsive to maternal trace mineral source and inclusion, but results are conflicting across studies. This is likely due to differences in basal forage mineral content and timing of treatments and liver sampling. Fetuses of beef heifers supplemented to meet Cu recommendations during the last two-thirds of gestation had greater liver Cu and Zn than fetuses of beef heifers deficient in dietary Cu (Fry et al., 2013). Calf liver Cu at birth, but not liver Zn or Mn, was also greater in calves born to beef heifers provided adequate dietary trace minerals and subcutaneous trace mineral injections throughout gestation compared with calves born to heifers that received saline injections (Stokes et al., 2018). Maternal supplementation above recommendations with organic, but not inorganic, Cu, Zn, and Mn in late gestation resulted in greater beef calf liver Cu and Zn, but not Mn, at birth than calves born to cows with no trace mineral supplementation (Marques et al., 2016), but calves born to cows fed the same inorganic and organic sources during mid- and late gestation had similar liver Cu, Zn, and Mn at birth and d 43 of age (Harvey et al., 2021a). Similarly, in the second year of a 2-yr study, calves born to cows supplemented an organic form of Cu in late gestation had greater liver Cu at 10 d of age than calves born to marginally Cu-deficient dams; however, there were no calf liver Cu differences in year 1 (Muehlenbein et al., 2001). Both Marques et al. (2016) and Muehlenbein et al. (2001) reported calves born to cows not supplemented and those born to cows supplemented with inorganic Cu had similar liver Cu concentrations. Calf liver Zn was not altered at 10 d of age by Zn supplementation compared with no supplementation in late gestational beef cows (Muehlenbein et al., 2001). These data collectively support the calf liver Cu differences between MMHAC and CON and lack of differences between ITM and CON in the current study. Moreover, some data support our hypothesis of greater liver Zn leading to maintenance of plasma Zn at 48 h, given Zn liver differences observed at birth in other studies. To our knowledge, no data are available to support or refute the hypothesis of altered perinatal Cu or Mn transfer when dams were provided both inorganic and chelated Cu together in late gestation.

Gestational treatment did not affect (*P *= 0.58) relative mRNA expression of metallothionein-1A in calf liver (Table 3). Metallothionein is a protein that can both store and donate Cu and Zn ions, making it essential in Cu and Zn metabolism (Cousins, 1985). It has been hypothesized that calf hepatic metallothionein is more responsive to Zn status than Cu status (López-Alonso et al., 2005); however, few data are available on the effects of gestational trace mineral supply on liver metallothionein in offspring, especially in livestock species. Fetuses collected in late gestation from Cu-deficient beef cows had similar metallothionein-1A expression compared with fetuses from Cu-adequate cows (Fry et al., 2013), and supplementation of organic vs. inorganic Cu, Zn, and Mn above recommendations during mid- and late gestation did not affect calf liver metallothionein (Harvey et al., 2021a). When rhesus monkeys were fed a Zn-deficient diet during gestation and the first month of lactation, neonatal liver metallothionein content at 30 d of age was similar to infants born to dams fed a Zn-adequate diet (Keen et al., 1989). However, rat pups born to Zn-deficient dams had less liver metallothionein than pups born to Zn-adequate dams (Gallant and Cherian, 1986). Hepatic metallothionein is responsive to dietary changes in Cu and Zn (Cousins, 1985); therefore, the lack of differences in the current study may be due to similar intake among treatments from colostrum and milk mineral (discussed in later section). Rat pups hepatically injected with Zn at 1 d of age had greater liver metallothionein content at days 2 and 7 of age, suggesting that neonatal liver metallothionein is responsive to greater Zn delivery (Chan and Cherian, 1993).

The age of calves at liver sampling should be taken into consideration because neonates mobilize Cu, Zn, and Mn stores in early life to manage the challenges that come with this stressful period (Aggett, 1998). Previously, liver metallothionein expression increased but liver Cu, Zn, and Mn decreased within the first 24 h of life (Harvey et al., 2021a) and liver Cu and Zn concentrations decreased from days 10 to 30 of age (Muehlenbein et al., 2001) in beef calves. Thus, age at sampling could explain inconsistencies of fetal and neonatal calf liver mineral results among different studies. Neonates also consume dietary trace minerals through colostrum and milk during this time, affecting the amount of trace mineral stores that need to be mobilized. In the current study, colostral Cu, Zn, and Mn concentration and content were not affected by gestational treatment (discussed in later section); therefore, calves were likely consuming similar amounts of these trace minerals. It may therefore be hypothesized that calf liver trace mineral differences were mainly due to differences in mineral accretion in late gestation in this study.

### Pregnant Cow Measures

#### Cow body weight and body condition score.

**Figure 2.**
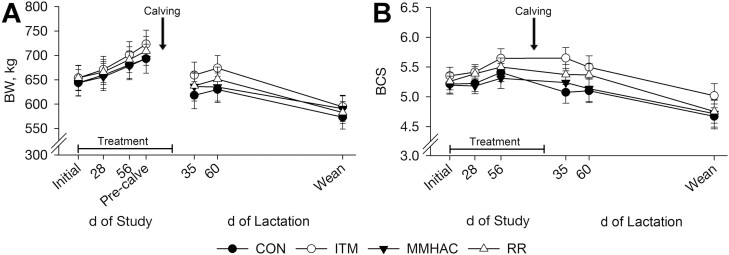
Effect of Cu, Zn, and Mn source and inclusion during late gestation on cow body weight (BW, Panel A) and body condition score (BCS, 1 = emaciated; 9 = obese, Panel B). Cows were individually-fed hay and supplemented with: no additional Cu, Zn, or Mn (control, CON), sulfate-based Cu, Zn, and Mn (inorganic, ITM) or methionine hydroxy analogue chelates of Cu, Zn, and Mn (MMHAC) to meet 133% of recommendations, or a combination of inorganic and chelated Cu, Zn, and Mn (reduce and replace, RR) to meet 100% of recommendations from 91.2 ± 6.2 d pre-calving until 11.0 ± 3.2 d post-calving. Study initiation = 91.2 ± 6.2 d pre-calving, pre-calving = 17.5 ± 8.0 d pre-calving (day 74 of study), weaning = 195.3 ± 8.3 d post-calving. Least square means ± SEM are presented (CON *n* = 11, ITM *n* = 11, MMHAC *n* = 10, RR *n* = 12). Gestational treatment did not affect BW (*P* = 0.74 to 0.98) or BCS (*P* = 0.13 to 0.88) at any time point.

Initial cow BW (Fig. 2A), BCS (Fig 2B), and age (Table 3) were similar (*P *≥ 0.87) among treatments. Throughout late pregnancy, gestational treatment did not affect (*P *≥ 0.47) cow BW or BCS. This was expected because, per study design, CP and NE_m_ intakes during late gestation were not affected (*P *≥ 0.51) by treatment (data not shown). In similar studies, gestational cow BW and BCS have generally not been affected by source or inclusion of these minerals (Muehlenbein et al., 2001; Ahola et al., 2004; Marques et al., 2016); however, cow performance was improved by organic trace mineral supplementation at some timepoints of another study (Harvey et al., 2021a). Typically, severe trace mineral deficiencies or toxicities (Graham, 1991; NASEM, 2016) cause impaired maintenance, growth, and production in beef cattle, but dietary trace minerals provided to cows in the current study and in previous studies discussed did not reach those extremes.

#### Cow metabolic and oxidative status

Initial cow plasma glucose, serum urea N, and serum NEFA were not different (*P *≥ 0.11), and gestational treatment did not affect (*P *≥ 0.35) these circulating metabolites during late gestation or at 1 h post-calving (Supplementary Table 1). This indicates that treatment did not affect cow metabolic status, further supporting the lack of differences in CP and NE_m_ intake, as well as cow BW and BCS.

Initial cow serum TBARS, GPx, GSH, GSSG, and GSH/GSSG ratio were not different (*P *≥ 0.11) among gestational treatments (Supplementary Table 2). Gestational treatment tended (*P *= 0.10) to affect serum TBARS, where MMHAC-fed cows had less (*P *= 0.01) TBARS than CON-fed cows during late gestation, but not at 1 h post-calving (*P* = 0.62). Gestational treatment did not affect (*P *≥ 0.13) cow serum GPx, GSH, GSSG, or GSH/GSSG ratio during late gestation or at 1 h post-calving. Thiobarbituric acid reactive substances are products of membrane phospholipid peroxidation by free radicals; therefore, elevated TBARS are a marker of increased oxidative stress for CON cows that were marginally deficient in Cu and Zn. Superoxide anion radical is one of the most common types of free radicals (Phaniendra et al., 2015) and is converted to hydrogen peroxide by superoxide dismutase, an enzyme containing Cu, Zn, or Mn depending on the cellular compartment (Fukai and Ushio-Fukai, 2011). Glutathione peroxidase, a selenoenzyme (Flohe et al., 1973), is responsible for converting hydrogen peroxide into water while converting GSH to GSSG (Weydert and Cullen, 2009). Thus, a reduction in GSH/GSSG ratio indicates greater oxidative stress. The discrepancies between TBARS and GSH/GSSG ratio results could be due to the presence of free radicals other than superoxide anion causing membrane damage. These results suggest that Cu, Zn, and Mn chelate supplementation may reduce oxidative stress in gestational beef cows. Yasui et al. (2014) reported transition dairy cows fed inorganic Cu, Zn, and Mn had less total antioxidant capacity and greater TBARS compared with cows fed a more bioavailable source. Oxidative stress markers were not affected in dairy cows provided daily boluses that contained inorganic or organic Cu, Zn, Mn, and Co from 30 d prepartum to 30 d postpartum (Osorio et al., 2016). These previous studies have more consistent oxidative stress data than the current study, which could be due to total antioxidant capacity encompassing more antioxidant enzymes than only GPx (Ghiselli et al., 2000).

#### Fetal and placental growth.

Gestational treatment did not affect (*P *≥ 0.28) calf BW or size measurements at birth of all calves or gestation length of AI-sired calves (Table 4). Typically, severe gestational trace mineral deficiencies result in impaired fetal growth, skeletal malformations, and neurological abnormalities if the deficiencies do not result in loss of pregnancy (Hidiroglou, 1980; Hurley, 1981; Hostetler et al., 2003). In the current study, CON diets were marginally deficient in Cu and Zn and generally sufficient for Mn, so impaired fetal growth was not expected (Graham, 1991). Although trace mineral injections (Stokes et al., 2018) and organic trace mineral boluses (Jacometo et al., 2015) in late gestation have affected fetal growth or calf size in early life in some studies, generally feeding trace minerals at or above recommendations during late gestation has not altered fetal growth (Stanton et al., 2000; Jacometo et al., 2015; Marques et al., 2016; Price et al., 2017; Harvey et al., 2021a).

**Table 4.** Effects of Cu, Zn, and Mn source and inclusion during late gestation on calf size and vigor at birth, transfer of passive immunity, and pre-weaning growth

Variable	Treatment^1^				SEM	*P*-value
	CON	ITM	MMHAC	RR		
Calf sex, % female	54.5	54.5	60.0	58.3	—	—
Gestation length^2^, d	283	284	282	283	1	0.70
Size at birth^3^						
Birth weight, kg	37.9	37.5	35.8	38.1	1.3	0.57
Shoulder to rump length, cm	57.7	58.3	57.8	57.8	1.0	0.97
Heart girth, cm	75.9	75.5	73.3	75.7	1.1	0.28
Abdominal girth, cm	72.4	70.4	70.1	71.4	1.4	0.59
Flank girth, cm	67.4	65.4	63.9	65.4	1.7	0.50
Cannon circumference, cm	12.1	12.2	12.2	12.2	0.2	0.97
Vigor at birth						
Time to stand^4^, min	19.1	25.1	18.6	29.4	4.3	0.13
Vigor score at 10 min^5^	3.07	3.11	3.09	3.36	0.17	0.54
48-h calf serum^6^						
Immunoglobulin G, mg/mL	47.9	42.9	39.5	42.8	4.7	0.57
Immunoglobulin A, mg/mL	2.67	3.21	3.84	2.61	0.95	0.68
Immunoglobulin M, mg/mL	2.42	2.13	2.42	1.69	0.51	0.61
Calf body weight^7^, kg						
Day 35	80.7	79.0	79.0	78.5	2.7	0.89
Day 60	108	110	107	104	3	0.54
Day 125	186	181	185	179	5	0.63
Weaning^8^	235	235	241	230	7	0.68
Pre-weaning ADG, kg/d	1.02	1.02	1.06	1.00	0.03	0.54

^1^Calves born to cows individually-fed hay and supplemented with: no additional Cu, Zn, or Mn (control, CON), sulfate-based Cu, Zn, and Mn (inorganic, ITM) or methionine hydroxy analogue chelates of Cu, Zn, and Mn (MMHAC) to meet 133% of recommendations, or a combination of inorganic and chelated Cu, Zn, and Mn (reduce and replace, RR) to meet 100% of recommendations from 91.2 ± 6.2 d pre-calving until 11.0 ± 3.2 d post-calving.

^2^Calves sired by artificial insemination only. CON *n* = 9, ITM *n* = 8, MMHAC *n* = 7, RR *n* = 8.

^3^CON *n* = 11, ITM *n* = 10, MMHAC *n* = 10, RR *n* = 12.

^4^Calf standing on all 4 feet for 5consecutive seconds. CON *n* = 8, ITM *n* = 9, MMHAC *n* = 8, RR *n* = 8.

^5^Scale ranging from 1 (very weak) to 5 (extremely vigorous). CON *n* = 10, ITM *n* = 8, MMHAC *n* = 10, RR *n* = 8.

^6^CON *n* = 11, ITM *n* = 8, MMHAC *n* = 9, RR *n* = 11.

^7^CON *n* = 11, ITM *n* = 11, MMHAC *n* = 9, RR *n* = 12.

^8^Calves were 195 ± 8 d of age at weaning.

Placental size parameters were not affected (*P *≥ 0.55) by gestational treatment (Table 5), which supports the lack of effect on calf size at birth. Few data are available on the effects of gestational trace mineral supplementation on ruminant placental size. In mice, pups born to dams fed a marginally Zn-deficient diet during gestation had lower placental weights compared with mice fed a Zn-adequate diet during gestation (Wilson et al., 2017). However, differences in placental types, fetal number, gestation length, and trace mineral recommendations between species make it difficult to compare these.

**Table 5.** Effects of Cu, Zn, and Mn source and inclusion during late gestation on placental size and minerals

Variable	Treatment^1^				SEM	*P*-value
	CON	ITM	MMHAC	RR		
Placental size^2^						
Dry cotyledonary weight, g	155	149	176	152	21	0.66
Dry intercotyledonary weight, g	162	174	179	193	19	0.55
Dry total placental weight, g	318	319	354	343	34	0.73
Number of cotyledons	76.9	71.7	77.3	71.7	10.5	0.94
Average dry cotyledonary weight, g	2.08	2.17	2.35	2.29	0.30	0.86
Average umbilical vessel diameter, mm	9.43	9.35	9.20	9.07	0.65	0.96
Cotyledon, mineral concentration^3^						
Cu, mg/kg dry matter	5.99	5.46	6.74	7.59	1.14	0.35
Zn, mg/kg dry matter	64.0	43.9	57.4	49.4	7.7	0.17
Mn, mg/kg dry matter	2.39	7.46	7.47	16.41	8.50	0.46
Cotyledon, mineral total^4^						
Cu, mg	0.962	0.857	1.226	1.124	0.264	0.64
Zn, mg	9.16	6.91	10.29	8.30	1.60	0.36
Mn, mg	0.822	1.260	1.488	1.313	0.423	0.58

^1^Cows were individually-fed hay and supplemented with: no additional Cu, Zn, or Mn (control, CON), sulfate-based Cu, Zn, and Mn (inorganic, ITM) or methionine hydroxy analogue chelates of Cu, Zn, and Mn (MMHAC) to meet 133% of recommendations, or a combination of inorganic and chelated Cu, Zn, and Mn (reduce and replace, RR) to meet 100% of recommendations from 91.2 ± 6.2 d pre-calving until 11.0 ± 3.2 d post-calving.

^2^CON *n* = 6, ITM *n* = 6, MMHAC *n* = 7, RR *n* = 8.

^3^Three large cotyledons subsampled per collected placenta. CON *n* = 6, ITM *n* = 5, MMHAC *n* = 7, RR *n* = 10.

^4^Cotyledonary mineral concentration multiplied by cotyledonary dry weight for placentas deemed complete. CON *n* = 6, ITM *n* = 5, MMHAC *n* = 7, RR *n* = 7.

Cotyledonary Cu, Zn, and Mn concentration and content were not affected (*P *≥ 0.17) by gestational treatment (Table 5). It is unclear if placental minerals are more indicative of placental accretion or placental transfer of Cu, Zn, and Mn prior to expulsion. Generally, total mineral content of the cotyledons has not been reported, as other studies cited here did not collect placental size data. Marques et al. (2016) observed cotyledonary Cu concentrations, but not Zn or Mn, to be greater in expelled placentas of beef cows supplemented organic trace minerals above recommendations during the last third of gestation compared with no trace mineral supplementation. Both Marques et al. (2016) and Harvey et al. (2021a) observed no change in cotyledonary Cu, Zn, and Mn between inorganic and organic sources of trace minerals during pregnancy. Placentomes collected from beef heifers fed a Cu-deficient diet during the last two-thirds of gestation had less Cu than placentomes collected from heifers fed a Cu-adequate diet (Fry et al., 2013). Cotyledonary and calf liver concentration treatment differences were consistent for Cu and Mn in several studies (Fry et al., 2013; Marques et al., 2016; Harvey et al., 2021a), but Zn was inconsistent in 2 of the studies (Fry et al., 2013; Marques et al., 2016). Although both maternal and calf liver Cu, along with calf liver Mn, were affected by gestational treatment, cotyledonary differences did not concur in the current study.

### Neonatal Calf and Colostrum Measures

#### Neonatal calf vigor.

Gestational treatment did not affect (*P *≥ 0.13) calf vigor measures (Table 4). Limited data are available on how maternal trace mineral nutrition affects neonatal ruminant vigor. Hansen et al. (2006) reported that calves born to beef heifers fed a Mn-deficient diet throughout gestation had greater incidence of unsteadiness or weakness at birth; however, late gestational ewe Fe, Cu, and Co supplementation did not affect lamb vigor at birth in another study (Norouzian et al., 2014). Our lab has previously reported that calves born in cold conditions of spring calving have poorer vigor than calves born in more thermoneutral conditions of fall calving (Wichman et al., 2022); thus, results may have differed if calves experienced cold stress.

#### Neonatal calf metabolic status.

Gestational treatment did not affect (*P *≥ 0.17) circulating energy metabolites (glucose, NEFA, or triglycerides), protein metabolites (urea N, creatinine, total protein, globulin, or albumin), liver enzymes (AST, CK, or GGT), anion gap, bicarbonate, or direct or total bilirubin in neonatal calves (Supplementary Table 3). In general, changes from 0 to 48 h follow our previous report of fall-born calf blood chemistry in Larson-Peine et al. (2022). Although trace minerals are important for metabolic functions (Spears, 1999), few data are available regarding the effects of maternal Cu, Zn, and Mn nutrition on neonatal ruminant offspring metabolic status. Serum albumin is the primary mode of Zn transportation in the blood (Giroux et al., 1976), so the lack of differences indicates albumin was not likely responsible for differences in neonatal calf plasma Zn. Previously, maternal supplementation with organic trace mineral boluses in late gestation resulted in calves with less circulating glucose and greater circulating urea N pre-suckling than calves whose dams received inorganic trace minerals, but there were no differences at 24 h post-colostrum consumption or in other serum chemistry values (Jacometo et al., 2015).

Gestational treatment did not affect (*P *≥ 0.12) serum macromineral Na, Cl, P, K, or Mg concentrations; however, the main effect of treatment did affect (*P *= 0.05) serum Ca. Calves born to cows fed MMHAC had greater (*P *≤ 0.05) serum Ca than all other treatments. These differences in neonatal serum Ca could be due to MMHAC calves receiving colostrum that had greater lactose compared with ITM and RR calves (discussed in later section). In adult humans, the inclusion of lactose in the diet resulted in delayed but prolonged dietary Ca absorption compared with those fed a lactose-deficient diet, which was hypothesized to be due to delayed gastric emptying (Cochet et al., 1983). In the current study, sampling time did not affect (*P *≥ 0.14) serum Ca of these calves, suggesting differences in lactose consumption may have persisted, or another mechanism may be at play.

Gestational treatment did not affect (*P *= 0.33) neonatal plasma cortisol (Supplementary Table 3), indicating that Cu, Zn, and Mn did not likely alter calf stress response at birth and 48 h. Limited data are available on gestational Cu, Zn, and Mn supply and neonatal circulating cortisol or stress response, but gestational trace mineral supplementation has altered cortisol in postweaning calves previously (Marques et al., 2016; Harvey et al., 2021b).

#### Colostrum yield and quality.

Gestational treatment tended (*P *≤ 0.10) to affect pre-suckling, single rear quarter colostrum volume and weight (Table 6). Cows fed MMHAC had greater (*P *≤ 0.05) colostrum volume than ITM and RR cows, but MMHAC volume was not different (*P *= 0.18) than CON. The same response was observed for colostrum weight, except that weight from MMHAC cows tended (*P *= 0.06) to be greater than ITM cows. We have previously demonstrated that the single most full rear quarter yield is a strong predictor of total colostrum yield in beef cows (*r*^2^ = 0.85; Rathert-Williams et al., 2023).

**Table 6.** Effects of Cu, Zn, and Mn source and inclusion during late gestation on colostrum and milk yield, micronutrients, and immunoglobulins (Ig)

Variable	Treatment^1^				SEM	*P*-values		
	CON	ITM	MMHAC	RR		Trt	Day	Trt × Day
Colostrum^2^								
Weight^2^, g	1,135^ab^	910^b^	1,552^a^	757^b^	259	0.10		
Volume^2^, mL	1,065^ab^	834^b^	1,481^a^	707^b^	247	0.09		
Mineral concentration								
Cu, µg/kg	166	203	188	167	27	0.61		
Zn, mg/kg	17.9	18.7	17.3	19.0	2.7	0.96		
Mn, µg/kg	34.8	43.6	54.8	42.6	10.3	0.48		
Mineral total^3^								
Cu, µg	210	201	346	151	74	0.21		
Zn, mg	16.9	16.5	20.7	12.9	3.3	0.32		
Mn, µg	58.2	50.5	118.2	43.6	28.9	0.19		
Ig concentration								
IgG, g/L	174	193	112	191	28	0.11		
IgA, g/L	8.49	12.03	7.60	10.88	2.80	0.55		
IgM, g/L	7.51	10.05	8.24	9.83	1.92	0.61		
Ig total^3^								
IgG, g	153	157	141	122	25	0.65		
IgA, g	7.06	8.82	6.48	6.89	1.99	0.79		
IgM, g	7.23	6.09	7.42	5.80	1.51	0.63		
Milk^4^								
Weight, kg/d						0.78	0.23	0.86
Day 35	10.16	9.88	10.40	10.16	0.80			
Day 60	10.03	9.38	10.18	9.25	0.62			
Volume, L/d						0.79	0.24	0.85
Day 35	10.20	9.93	10.43	10.19	0.79			
Day 60	10.11	9.41	10.21	9.30	0.64			
Mineral concentration								
Cu, µg/kg						0.52	0.21	0.19
Day 35	85.5	81.9	77.8	147.3	31.2			
Day 60	82.8	74.5	87.8	67.2	12.3			
Zn, mg/kg						0.54	0.001	0.30
Day 35	2.98	2.82	2.94	3.11	0.23			
Day 60	2.62	2.27	2.85	2.37	0.23			
Mn, µg/kg						0.52	0.07	0.19
Day 35	60.3	46.5	49.0	95.6	21.7			
Day 60	41.3	46.5	46.8	36.8	6.8			
Mineral total^5^								
Cu, µg						0.57	0.17	0.18
Day 35	900	834	870	1,584	366			
Day 60	840	678	1,002	630	162			
Zn, mg						0.53	0.004	0.29
Day 35	30.3	29.0	30.7	33.2	3.8			
Day 60	26.5	21.5	29.8	22.3	2.7			
Mn, µg						0.64	0.05	0.26
Day 35	640	510	549	1,023	255			
Day 60	415	439	489	347	90			

^1^Cows were individually-fed hay and supplemented with: no additional Cu, Zn, or Mn (control, CON), sulfate-based Cu, Zn, and Mn (inorganic, ITM) or methionine hydroxy analogue chelates of Cu, Zn, and Mn (MMHAC) to meet 133% of recommendations, or a combination of inorganic and chelated Cu, Zn, and Mn (reduce and replace, RR) to meet 100% of recommendations from 91.2 ± 6.2 d pre-calving until 11.0 ± 3.2 d post-calving.

^2^A single, rear quarter of colostrum was completely milked pre-suckling (57.5 ± 17.5 min post-calving). CON *n* = 10, ITM *n* = 9, MMHAC *n* = 7, RR *n* = 9.

^3^Total from a single rear quarter, pre-suckling.

^4^Daily milk production was estimated using 4-h milk yield multiplied by 6. Day 35 of lactation: CON *n* = 11, ITM *n* = 11, MMHAC *n* = 9, RR *n* = 11. Day 60 of lactation: CON *n* = 11, ITM *n* = 10, MMHAC *n* = 9, RR *n* = 10.

^5^Total from 24-h estimated milk yield.

^a,b^Within an item, treatment means differ (*P* < 0.10).

Observed differences in colostrum yield were not expected. Although previous colostrum yields of these cows were unknown, typical factors that affect colostrum yield including age, BCS, and intakes of energy and protein (McGee and Earley, 2019) were similar among treatments. To our knowledge, effects of trace mineral supplementation on colostrum yield have not been greatly studied. Karkoodi et al. (2012) observed that source (different proportions of inorganic and organic to meet recommendations) of Cu, Zn, Mn, and Se supplementation in the last 3 wk of pregnancy did not alter pre-suckling colostrum yield in dairy cows, but this may not have been implemented early enough in gestation to detect colostrum yield differences. In another study, glycine salts of Cu, Zn, and Mn during the dry period increased dairy cow colostrum yield compared with controls who were not supplemented (Roshanzamir et al., 2020). Supranutritional Se during mid- and late gestation has been reported to increase colostrum yields in first parity ewes, although these ewes also had greater average daily gain and BCS which may have contributed (Meyer et al., 2011). Overall, more research is necessary to determine the importance of trace mineral supplementation for optimal colostrum yield.

Gestational treatment did not affect (*P *≥ 0.19) colostral Cu, Zn, and Mn concentration or content (Table 6). Trace mineral sequestration by the mammary gland and secretion into the alveoli lumen is highly regulated; therefore, major dietary changes are required to alter Cu, Zn, and Mn concentrations in milk (Lönnerdal et al., 1981; Kelleher and Lönnerdal, 2005). This regulation likely also affects trace mineral incorporation into colostrum. Previously, trace mineral source and inclusion during gestation have affected colostral Cu and/or Zn concentrations in dairy cows (Kincaid and Socha, 2004; Kinal et al., 2007) and Zn in beef cows (Price et al., 2017). Colostral Cu, Zn, and Mn were not affected by gestational trace mineral nutrition in other studies (Muehlenbein et al., 2001; Harvey et al., 2021a). Differences in maternal mineral status may have affected the extent to which endogenous trace mineral stores could be mobilized to deliver similar trace minerals to the mammary gland.

Gestational treatment affected (*P* = 0.02) colostral protein concentration and tended (*P* ≤ 0.10) to affect lactose and triglyceride concentrations, but treatment did not affect (*P *> 0.67) urea N concentration (Supplementary Table 4). Colostral protein concentration was greater (*P* = 0.003) in RR cows than MMHAC and tended (*P* = 0.08) to be greater for RR than CON cows. Protein concentration was also greater (*P* = 0.009) in ITM cows than MMHAC cows, but MMHAC cows were not different (*P* = 0.14) from CON cows. Colostral lactose concentration was greater (*P *≤ 0.04) in MMHAC cows than ITM and RR but was not different (*P *= 0.18) than CON cows. Cows fed RR had greater (*P *≤ 0.04) triglyceride concentration compared with CON and MMHAC cows but were not different (*P *= 0.27) than ITM cows. Total colostral lactose was greater (*P *≤ 0.03) in MMHAC cows than ITM and RR but was not different (*P *= 0.13) than CON. Gestational treatment did not affect (*P *≥ 0.18) total colostral triglycerides, protein, or urea N. Differences in macronutrients were not expected, as gestational metabolic status was not affected by late gestational trace mineral treatment. However, colostral lactose may explain differences in colostrum yield given the role of lactose as the primary osmole, driving water secretion into milk (Linzell and Peaker, 1971). As total colostral triglycerides and protein were not different among treatments, the colostral triglyceride and protein concentration results could be driven by RR cows yielding less colostrum, resulting in more concentrated colostrum. Trace mineral source and inclusion during late gestation did not affect macronutrient composition of pre-suckling colostrum in either dairy (Karkoodi et al., 2012; Roshanzamir et al., 2020) or beef cows (Price et al., 2017) previously. However, Kinal et al. (2007) reported greater colostral lactose concentrations in dairy cows fed inorganic Cu, Zn, and Mn compared with cows fed a diet including just organic (chelates) or both organic and inorganic Cu, Zn, and Mn. They also reported greater colostral crude fat concentration in cows supplemented both inorganic and organic or just organic trace mineral compared with inorganic (Kinal et al., 2007). These results are somewhat contradictory of the current study and highlight that further investigation is needed into trace mineral supplementation’s role in colostral nutrient synthesis, especially for lactose.

#### Transfer of passive immunity.

Colostral IgG, IgA, and IgM concentration and content were not affected (*P *≥ 0.11) by gestational treatment (Table 6). Although concentration of IgG, the major Ig isoform in bovine colostrum, approached a tendency (*P* = 0.11), this was likely due to dilution from colostrum yield given the total IgG content. Organic Cu, Zn, and Mn supplementation during late gestation resulted in greater colostral IgG concentration in dairy cows (Kincaid and Socha, 2004) and IgM concentration in beef cows (Price et al., 2017), but colostral Ig concentrations have also been unaffected by trace mineral source in beef cows (Muehlenbein et al., 2001) and dairy cows (Karkoodi et al., 2012; Jacometo et al., 2015). Total content of Ig is likely more important, but rarely determined due to the lack of colostrum yield determination. This variation in results indicates that the relationship between gestational trace mineral supply and colostral Ig concentration is complex and further investigation is needed.

Gestational treatment did not affect (*P *≥ 0.57) calf serum IgG, IgA, or IgM concentrations at 48 h of age (Table 4). The range of serum IgG was 15.6 to 77.5 mg/mL, which are all well above the minimum threshold of 8 to 9 mg/mL for beef calves set by Todd et al. (2018). Two calves (1 MMHAC and 1 RR) had serum IgG below the 24 mg/mL threshold set by Waldner and Rosengren (2009). Minimal morbidity was observed other than one calf (ITM) that was treated for severe dehydration due to scours at 9 d of age, and one calf (RR) who was treated for respiratory disease symptoms at 145 d of age. All calves survived to weaning, suggesting that passive transfer was adequate in this study.

In the current study, calf vigor was also not affected by gestational treatment, which suggests that calf colostrum consumption was likely similar among treatments. However, previous studies suggest colostral Ig is not always analogous with calf serum Ig; therefore, there may be other factors contributing to these results such as differences in Ig absorption capacity. Boland and others (2005) reported lambs born to ewes consuming high concentrations of trace minerals had less IgG absorption compared with lambs born to control ewes, even if those lambs received colostrum from control dams, suggesting that trace minerals supplied to the fetus can affect Ig absorption capacity. Neonatal beef calf serum Ig changes have been observed that do not agree with colostral Ig, where serum IgG was affected by Cu source and inclusion during gestation (Muehlenbein et al., 2001), and serum IgA were affected by Cu, Zn, and Mn in late gestation (Price et al., 2017).

### Lactating Cow and Pre-weaning Calf Measures

#### Cow performance and oxidative status.

Gestational treatment did not affect (*P *≥ 0.13) cow BW or BCS during lactation or at weaning (Fig. 2). This was expected as late gestational cow performance was not affected during the treatment period, and cows were managed as a single group from treatment termination to weaning.

During lactation, serum TBARS tended (*P *= 0.10) to be affected by the interaction of gestational treatment × sampling day (Supplementary Table 2), where cows fed RR during late gestation had greater (*P *= 0.04) TBARS at day 35 of lactation than CON cows. At day 60 of lactation, MMHAC-fed cows had greater (*P *≤ 0.05) serum TBARS than CON and ITM, and MMHAC tended (*P *= 0.09) to have greater TBARS than RR. Despite this, cow serum GPx, GSH, GSSG, and GSH/GSSG ratio during lactation were not affected (*P *≥ 0.20) by gestational treatment. In the current study, greater circulating TBARS in cows fed MMHAC during lactation was unexpected because MMHAC cows had reduced TBARS during the treatment period. Lactational data were collected after treatment termination when cows had ad libitum access to an inorganic trace mineral supplement that could have led to variable trace mineral intakes and the inconsistencies in these results.

#### Milk yield and quality.

Milk yields (weight and volume) at days 35 and 60 of lactation were not affected (*P *≥ 0.78) by gestational treatment (Table 6). Although trace mineral supplementation of dairy cows starting in late gestation and continuing into lactation resulted in variable milk yield treatment differences during the treatment period in previous studies (Yasui et al., 2014; Osorio et al., 2016; Roshanzamir et al., 2020; Mion et al., 2022), gestational treatments alone are rarely studied. Because trace mineral treatments were terminated 11 d post-calving in the current study, similar milk yields at days 35 and 60 of lactation were expected. In agreement with the current study, Harvey et al. (2021a) reported no effect of gestational trace mineral source at day 42 of lactation using the weigh-suckle-weigh method in beef cows.

Gestational treatment did not affect (*P* ≥ 0.18) milk Cu, Zn, or Mn concentration or total content (Table 6), or milk lactose, triglycerides, protein, and urea N concentration and content (Supplementary Table 4) in this study. A lack of differences is likely due to milk samplings occurring after treatment termination. Source of Cu, Zn, and Mn during late gestation did not affect mineral composition of milk in beef (Harvey et al., 2021a) or dairy cows (Krys et al., 2009). Supplementing dairy cows with different sources of trace minerals during late gestation and lactation has resulted in variable milk macronutrient yield differences (Osorio et al., 2016), but generally no effects are observed even when mineral supplementation treatments continue into lactation (Yasui et al., 2014; Roshanzamir et al., 2020; Mion et al., 2022).

#### Pre-weaning calf growth and metabolic status.

Gestational treatment did not affect (*P *≥ 0.54) pre-weaning BW or growth (Table 4). In previous studies, calf weaning BW or pre-weaning average daily gain was not affected by trace mineral supply during gestation (Stokes et al., 2018; Harvey et al., 2021a), lactation (Olson et al., 1999), or gestation and lactation (Muehlenbein et al., 2001; Sprinkle et al., 2006). However, supplementation of organic trace minerals during late gestation (Marques et al., 2016) or during gestation and lactation (Stanton et al., 2000; Price et al., 2017) resulted in greater calf weaning BW compared with calves born to dams that received no trace mineral supplement or inorganic trace mineral supplement, respectively. Conversely, calves born to cows that received trace mineral supplementation during late gestation and lactation had lower weaning BW than calves born to cows that received no trace mineral supplement in both years of a 2-yr study (Ahola et al., 2004). There are many differences among these studies, such as length and source of supplementation, basal diet trace mineral content, calf pre-weaning health status, and calf age at weaning, which likely caused variability in these results. However, in the current study, pre-weaning calf growth was expected to be similar among treatments because there were no differences in calf birth BW, milk yield or composition, and calf health after treatment termination at 11 d post-calving. Greater disease occurrence may have affected results, especially during early life due to calf mineral status differences.

Pre-weaning calf circulating glucose, serum urea N, and NEFA were not affected (*P *≥ 0.46) by gestational treatment (Supplementary Table 5). These data support the lack of pre-weaning calf growth treatment differences. Trace minerals are involved in many metabolic pathways (Spears, 1999), but maternal trace mineral supplementation did not affect neonatal metabolic status and treatments ceased when calves were 11 d of age; therefore, it was unlikely that metabolic status would be affected later in life.

## Conclusion

Inclusion of chelated Cu, Zn, and Mn to supply 133% of recommendations in the diet of late gestation beef cows resulted in improved maternal Cu status, and inclusion of chelated Cu, Zn, and Mn, regardless of amount, in maternal diets improved neonatal calf Cu status. Neonatal calf plasma Zn was maintained when Cu, Zn, and Mn were supplied to 133% of recommendations during late gestation. Marginal Cu and Zn deficiency during late gestation resulted in greater circulating oxidative stress markers during gestation but not during lactation, and chelated Cu, Zn, and Mn resulted in greater colostrum yield and altered macronutrients, but not micronutrients, in colostrum. Fetal growth, calf vigor, and pre-weaning growth were not affected by late gestational trace mineral treatment. Beef cows in this study were generally in adequate Cu, Zn, and Mn status at treatment initiation, which likely affected results. There were multiple discrepancies among cow and calf mineral status results, suggesting perinatal trace mineral transfer is complex and unclear. Further investigation to determine gestational trace mineral recommendations of beef females and neonatal calves is necessary to better understand trace mineral transfer and production responses, especially in females that enter late gestation in poor trace mineral status.

## Supplementary Material

txad097_suppl_Supplementary_Tables
