# Supplementary material for: Effects of copper, zinc, and manganese source and inclusion during late gestation on beef cow–calf performance, mineral transfer, and metabolism
Source: Transl Anim Sci. 2023 Aug 16;7(1):txad097. doi: 10.1093/tas/txad097 (PMC10519816; doi:10.1093/tas/txad097)
Supplement: txad097_suppl_Supplementary_Tables [file txad097_suppl_supplementary_tables.docx]

| Supplementary Table 1. Effects of Cu, Zn, and Mn source and inclusion during late gestation on cow circulating glucose, urea N, and non-esterified fatty acids (NEFA) during gestation and 1 h post-calving | | | | | | | | | |
| --- | --- | --- | --- | --- | --- | --- | --- | --- | --- |
|  | Treatment^1^ | | | |  | *P*-values | | | |
| Variable | CON | ITM | MMHAC | RR | SEM | Trt | Day | Trt × Day |  |
| Gestation^2^ |  |  |  |  |  |  |  |  |  |
| Plasma glucose, mg/dL | |  |  |  |  | 0.61 | 0.02 | 0.82 |  |
| Initial^3^ | 70.9 | 69.7 | 73.3 | 69.3 | 1.8 |  |  |  |  |
| d 28 of study | 65.8 | 66.6 | 68.2 | 67.4 | 1.8 |  |  |  |  |
| d 56 of study | 70.3 | 67.4 | 67.2 | 68.6 | 1.9 |  |  |  |  |
| Pre-calving^4^ | 70.1 | 68.2 | 68.7 | 67.9 | 1.9 |  |  |  |  |
| Serum urea N, mg/dL |  |  |  |  |  | 0.97 | 0.02 | 0.95 |  |
| Initial^3^ | 5.72 | 5.49 | 5.42 | 5.82 | 0.56 |  |  |  |  |
| d 28 of study | 6.29 | 6.10 | 6.25 | 6.20 | 0.30 |  |  |  |  |
| d 56 of study | 6.88 | 6.80 | 7.27 | 6.48 | 0.43 |  |  |  |  |
| Pre-calving^4^ | 6.52 | 6.59 | 6.43 | 6.57 | 0.24 |  |  |  |  |
| Serum NEFA, µEq/L |  |  |  |  |  | 0.54 | <0.001 | 0.88 |  |
| Initial^3^ | 570 | 475 | 548 | 601 | 88 |  |  |  |  |
| d 28 of study | 392 | 380 | 338 | 334 | 50 |  |  |  |  |
| d 56 of study | 448 | 346 | 361 | 383 | 45 |  |  |  |  |
| Pre-calving^4^ | 446 | 357 | 445 | 417 | 67 |  |  |  |  |
|  |  |  |  |  |  |  |  |  |  |
| 1 h post-calving^5^ |  |  |  |  |  |  |  |  |  |
| Plasma glucose, mg/dL | 101.8 | 95.9 | 91.7 | 95.9 | 9.5 | 0.85 |  |  |  |
| Serum urea N, mg/dL | 6.03 | 6.44 | 5.68 | 6.27 | 0.35 | 0.35 |  |  |  |
| Serum NEFA, µEq/L | 775 | 679 | 742 | 853 | 91 | 0.40 |  |  |  |
| ^1^Cows were individually-fed hay and supplemented with: no additional Cu, Zn, or Mn (control, CON), sulfate-based Cu, Zn, and Mn (inorganic, ITM) or methionine hydroxy analogue chelates of Cu, Zn, and Mn (MMHAC) to meet 133% of recommendations, or a combination of inorganic and chelated Cu, Zn, and Mn (reduce and replace, RR) to meet 100% of recommendations from 91.2 ± 6.2 d pre-calving until 11.0 ± 3.2 d post-calving.  ^2^CON *n* = 11, ITM *n* = 11, MMHAC *n* = 10, RR *n* = 12.  ^3^Initial = 91.2 ± 6.2 d pre-calving.  ^4^Pre-calving = 17.5 ± 8.0 d pre-calving, d 74 of study (average).  ^5^CON *n* = 11, ITM *n* = 9, MMHAC *n* = 7, RR *n* = 10. | | | | | | | | | |

| Supplementary Table 2. Effects of Cu, Zn, and Mn source and inclusion during late gestation on cow serum thiobarbituric acid reactive substances (TBARS), glutathione peroxidase (GPx), reduced (GSH) and oxidized glutathione (GSSG), and GSH/GSSG ratio | | | | | | | | | | |  |
| --- | --- | --- | --- | --- | --- | --- | --- | --- | --- | --- | --- |
|  | Treatment^1^ | | | | |  | | *P*-values | | | |
| Variable | CON | ITM | MMHAC | RR | SEM | | Trt | | Day | Trt × Day | |
| Gestation^2^ |  |  |  |  |  | |  | |  |  | |
| TBARS^3^, µM | 7.20^a^ | 6.87^ab^ | 6.48^b^ | 6.82^ab^ | 0.20 | | 0.10 | | <0.001 | 0.93 | |
| Initial^4^ | 9.21 | 8.97 | 8.48 | 8.74 | 0.52 | |  | |  |  | |
| d 28 of study | 6.74 | 6.34 | 6.27 | 7.03 | 0.39 | |  | |  |  | |
| d 56 of study | 5.92 | 5.40 | 5.04 | 5.64 | 0.24 | |  | |  |  | |
| Pre-calving^5^ | 6.90 | 6.76 | 6.12 | 5.88 | 0.58 | |  | |  |  | |
| GPx, mg/g protein |  |  |  |  |  | | 0.64 | | 0.04 | 0.13 | |
| Initial^4^ | 2.74 | 2.51 | 2.64 | 2.78 | 0.16 | |  | |  |  | |
| d 28 of study | 2.93 | 2.62 | 2.84 | 2.82 | 0.14 | |  | |  |  | |
| d 56 of study | 2.68 | 2.54 | 2.76 | 2.54 | 0.16 | |  | |  |  | |
| Pre-calving^5^ | 2.69 | 2.71 | 2.91 | 2.37 | 0.20 | |  | |  |  | |
| GSH, µg/g protein |  |  |  |  |  | | 0.76 | | 0.01 | 0.48 | |
| Initial^4^ | 94.4 | 124.9 | 66.6 | 68.4 | 27.6 | |  | |  |  | |
| d 28 of study | 62.4 | 75.7 | 51.4 | 60.5 | 13.9 | |  | |  |  | |
| d 56 of study | 68.0 | 75.2 | 55.7 | 62.6 | 17.6 | |  | |  |  | |
| Pre-calving^5^ | 67.6 | 62.1 | 77.5 | 75.9 | 17.4 | |  | |  |  | |
| GSSG, ng/g protein |  |  |  |  |  | | 0.29 | | <0.001 | 0.87 | |
| Initial^4^ | 181 | 149 | 175 | 160 | 17 | |  | |  |  | |
| d 28 of study | 205 | 183 | 199 | 179 | 15 | |  | |  |  | |
| d 56 of study | 140 | 112 | 153 | 108 | 20 | |  | |  |  | |
| Pre-calving^5^ | 128 | 104 | 141 | 90 | 21 | |  | |  |  | |
| GSH/GSSG ratio^6^ |  |  |  |  |  | | 0.77 | | <0.001 | 0.48 | |
| Initial^4^ | 606 | 888 | 466 | 530 | 190 | |  | |  |  | |
| d 28 of study | 366 | 433 | 308 | 451 | 109 | |  | |  |  | |
| d 56 of study | 644 | 854 | 496 | 857 | 231 | |  | |  |  | |
| Pre-calving^5^ | 869 | 817 | 809 | 1,123 | 339 | |  | |  |  | |
|  |  |  |  |  |  | |  | |  |  | |
| 1 h post-calving^7^ |  |  |  |  |  | |  | |  |  | |
| TBARS, µM | 8.09 | 6.87 | 7.45 | 7.26 | 0.83 | | 0.62 | |  |  | |
| GPx, mg/g protein | 2.32 | 2.23 | 2.56 | 2.24 | 0.28 | | 0.78 | |  |  | |
| GSH, µg/g protein | 38.1 | 22.1 | 26.5 | 32.7 | 12.3 | | 0.69 | |  |  | |
| GSSG, ng/g protein | 162 | 120 | 184 | 143 | 25 | | 0.24 | |  |  | |
| GSH/GSSG ratio^6^ | 300 | 218 | 186 | 239 | 115 | | 0.85 | |  |  | |
|  |  |  |  |  |  | |  | |  |  | |
| Lactation^8^ |  |  |  |  |  | |  | |  |  | |
| TBARS, µM |  |  |  |  |  | | 0.26 | | 0.45 | 0.10 | |
| d 35 | 5.62^y^ | 6.05^xy^ | 5.89^xy^ | 6.68^x^ | 0.41 | |  | |  |  | |
| d 60 | 5.53^y^ | 5.56^y^ | 6.69^x^ | 5.74^y^ | 0.42 | |  | |  |  | |
| GPx, mg/g protein |  |  |  |  |  | | 0.40 | | <0.001 | 0.34 | |
| d 35 | 2.51 | 1.95 | 2.38 | 2.19 | 0.25 | |  | |  |  | |
| d 60 | 1.44 | 1.19 | 1.57 | 1.30 | 0.21 | |  | |  |  | |
| GSH, µg/g protein |  |  |  |  |  | | 0.20 | | 0.13 | 0.65 | |
| d 35 | 42.4 | 24.9 | 23.1 | 18.1 | 9.4 | |  | |  |  | |
| d 60 | 40.0 | 34.1 | 33.8 | 25.1 | 7.8 | |  | |  |  | |
| GSSG, ng/g protein |  |  |  |  |  | | 0.28 | | <0.001 | 0.91 | |
| d 35 | 194 | 156 | 199 | 165 | 22 | |  | |  |  | |
| d 60 | 155 | 119 | 150 | 116 | 22 | |  | |  |  | |
| GSH/GSSG ratio^6^ |  |  |  |  |  | | 0.73 | | <0.001 | 0.64 | |
| d 35 | 264 | 166 | 186 | 124 | 63 | |  | |  |  | |
| d 60 | 338 | 368 | 328 | 280 | 99 | |  | |  |  | |
| ^1^Cows were individually-fed hay and supplemented with: no additional Cu, Zn, or Mn (control, CON), sulfate-based Cu, Zn, and Mn (inorganic, ITM) or methionine hydroxy analogue chelates of Cu, Zn, and Mn (MMHAC) to meet 133% of recommendations, or a combination of inorganic and chelated Cu, Zn, and Mn (reduce and replace, RR) to meet 100% of recommendations from 91.2 ± 6.2 d pre-calving until 11.0 ± 3.2 d post-calving.  ^2^CON *n* = 11, ITM *n* = 11, MMHAC *n* = 10, RR *n* = 12.  ^3^Treatment means listed above interactive means due to presence of treatment main effect.  ^4^Initial = 91.2 ± 6.2 d pre-calving.  ^5^Pre-calving = 17.5 ± 8.0 d pre-calving, d 74 of study (average).  ^6^Ratio of GSH (ng) to GSSG (ng).  ^7^CON *n* = 11, ITM *n* = 9, MMHAC *n* = 7, RR *n* = 10.  ^8^CON *n* = 11, ITM *n* = 11, MMHAC *n* = 9, RR *n* = 12.  ^a,b^Within an item, treatment means differ (*P* < 0.10).  ^x,y^Within an item, interative means differ (*P* < 0.10). | | | | | | | | | | |  |

| Supplementary Table 3. Effects of Cu, Zn, and Mn source and inclusion during late gestation on calf serum chemistry and cortisol concentrations at 0 and 48 h of age^1^ | | | | | | | | |  |
| --- | --- | --- | --- | --- | --- | --- | --- | --- | --- |
|  | Treatment^2^ | | | |  | *P*-value | | | |
| Variable | CON | ITM | MMHAC | RR | SEM | Trt | Hour | Trt × Hour | |
| Glucose, mg/dL |  |  |  |  |  | 0.52 | <0.001 | 0.63 | |
| 0 h | 42 | 38 | 45 | 48 | 6 |  |  |  | |
| 48 h | 119 | 122 | 130 | 119 | 7 |  |  |  | |
| Non-esterified fatty acids, µEq/L |  |  |  |  |  | 0.26 | 0.02 | 0.17 | |
| 0 h | 469 | 453 | 214 | 480 | 104 |  |  |  | |
| 48 h | 235 | 258 | 283 | 332 | 34 |  |  |  | |
| Triglycerides^3^, mg/L |  |  |  |  |  | 0.75 | <0.001 | 0.86 | |
| 0 h | 92 | 92 | 58 | 102 | 18 |  |  |  | |
| 48 h | 656 | 734 | 695 | 772 | 105 |  |  |  | |
| Urea N, mg/dL |  |  |  |  |  | 0.51 | 0.002 | 0.26 | |
| 0 h | 6.18 | 6.37 | 6.57 | 6.03 | 0.39 |  |  |  | |
| 48 h | 10.27 | 7.32 | 7.57 | 8.46 | 1.40 |  |  |  | |
| Creatinine, mg/dL |  |  |  |  |  | 0.46 | <0.001 | 0.53 | |
| 0 h | 3.91 | 4.29 | 4.90 | 4.22 | 0.47 |  |  |  | |
| 48 h | 1.15 | 1.11 | 1.18 | 1.07 | 0.06 |  |  |  | |
| Total protein, g/dL |  |  |  |  |  | 0.93 | <0.001 | 0.65 | |
| 0 h | 4.29 | 4.23 | 4.40 | 4.37 | 0.08 |  |  |  | |
| 48 h | 6.94 | 7.28 | 7.02 | 6.92 | 0.32 |  |  |  | |
| Globulin, g/dL |  |  |  |  |  | 0.79 | <0.001 | 0.50 | |
| 0 h | 1.43 | 1.39 | 1.54 | 1.54 | 0.05 |  |  |  | |
| 48 h | 4.59 | 5.09 | 4.68 | 4.61 | 0.34 |  |  |  | |
| Albumin, g/dL |  |  |  |  |  | 0.53 | <0.001 | 0.71 | |
| 0 h | 2.89 | 2.86 | 2.89 | 2.87 | 0.05 |  |  |  | |
| 48 h | 2.38 | 2.27 | 2.39 | 2.37 | 0.06 |  |  |  | |
| Aspartate aminotransferase, U/L |  |  |  |  |  | 0.74 | <0.001 | 0.82 | |
| 0 h | 16.7 | 15.1 | 14.1 | 14.9 | 1.1 |  |  |  | |
| 48 h | 47.4 | 44.5 | 46.0 | 47.4 | 2.9 |  |  |  | |
| Creatine kinase, U/L |  |  |  |  |  | 0.23 | 0.66 | 0.50 | |
| 0 h | 150 | 82 | 80 | 108 | 32 |  |  |  | |
| 48 h | 120 | 82 | 108 | 138 | 21 |  |  |  | |
| Gamma glutamyl transpeptidase, U/L |  |  |  |  |  | 0.19 | <0.001 | 0.19 | |
| 0 h | 13 | 9 | 11 | 11 | 1 |  |  |  | |
| 48 h | 717 | 829 | 1,219 | 653 | 213 |  |  |  | |
| Anion gap, mEq/L |  |  |  |  |  | 0.63 | <0.001 | 0.47 | |
| 0 h | 21.6 | 21.4 | 23.2 | 22.5 | 1.2 |  |  |  | |
| 48 h | 19.4 | 19.2 | 19.4 | 17.8 | 0.7 |  |  |  | |
| Bicarbonate, mEq/L |  |  |  |  |  | 0.68 | 0.06 | 0.31 | |
| 0 h | 28.6 | 28.6 | 26.6 | 27.2 | 0.8 |  |  |  | |
| 48 h | 26.0 | 26.9 | 26.9 | 26.7 | 0.9 |  |  |  | |
| Direct bilirubin, mg/dL |  |  |  |  |  | 0.51 | 0.04 | 0.47 | |
| 0 h | 0.112 | 0.122 | 0.102 | 0.133 | 0.012 |  |  |  | |
| 48 h | 0.121 | 0.138 | 0.168 | 0.157 | 0.028 |  |  |  | |
| Total bilirubin, mg/dL |  |  |  |  |  | 0.43 | 0.04 | 0.32 | |
| 0 h | 0.308 | 0.351 | 0.271 | 0.346 | 0.032 |  |  |  | |
| 48 h | 0.317 | 0.367 | 0.430 | 0.439 | 0.067 |  |  |  | |
| Sodium, mEq/L |  |  |  |  |  | 0.57 | <0.001 | 0.29 | |
| 0 h | 146 | 146 | 144 | 145 | 1 |  |  |  | |
| 48 h | 140 | 140 | 140 | 139 | 1 |  |  |  | |
| Calcium^4^, mg/dL | 11.4^b^ | 11.3^b^ | 11.8^a^ | 11.4^b^ | 0.2 | 0.05 | 0.14 | 0.68 | |
| 0 h | 11.2 | 11.2 | 11.7 | 11.4 | 0.2 |  |  |  | |
| 48 h | 11.5 | 11.3 | 11.9 | 11.4 | 0.2 |  |  |  | |
| Chloride, mEq/L |  |  |  |  |  | 0.45 | 0.01 | 0.51 | |
| 0 h | 102 | 102 | 100 | 101 | 1 |  |  |  | |
| 48 h | 100 | 99 | 99 | 100 | 1 |  |  |  | |
| Phosphorus, mg/dL |  |  |  |  |  | 0.60 | 0.66 | 0.44 | |
| 0 h | 7.48 | 7.38 | 7.73 | 7.68 | 0.35 |  |  |  | |
| 48 h | 8.10 | 7.42 | 7.85 | 7.29 | 0.38 |  |  |  | |
| Potassium, mEq/L |  |  |  |  |  | 0.12 | 0.97 | 0.63 | |
| 0 h | 5.37 | 5.31 | 5.32 | 5.09 | 0.14 |  |  |  | |
| 48 h | 5.54 | 5.22 | 5.19 | 5.12 | 0.15 |  |  |  | |
| Magnesium, mg/dL |  |  |  |  |  | 0.23 | 0.03 | 0.20 | |
| 0 h | 2.25 | 2.37 | 2.48 | 2.38 | 0.09 |  |  |  | |
| 48 h | 2.29 | 2.13 | 2.41 | 2.22 | 0.09 |  |  |  | |
| Cortisol, μg/dL |  |  |  |  |  | 0.33 | <0.001 | 0.42 | |
| 0 h | 12.6 | 12.0 | 14.0 | 12.1 | 0.8 |  |  |  | |
| 48 h | 2.2 | 2.2 | 2.2 | 2.0 | 0.4 |  |  |  | |
| ^1^At 0 h: CON *n* = 11, ITM *n* = 10, MMHAC *n* = 10, RR *n* = 10. At 48 h: CON *n* = 11, ITM *n* = 8, MMHAC *n* = 9, RR *n* = 11.  ^2^Calves born to cows individually-fed hay and supplemented with: no additional Cu, Zn, or Mn (control, CON), sulfate-based Cu, Zn, and Mn (inorganic, ITM) or methionine hydroxy analogue chelates of Cu, Zn, and Mn (MMHAC) to meet 133% of recommendations, or a combination of inorganic and chelated Cu, Zn, and Mn (reduce and replace, RR) to meet 100% of recommendations from 91.2 ± 6.2 d pre-calving until 11.0 ± 3.2 d post-calving.  ^3^Triglycerides were measured in plasma.  ^4^Treatment means listed above interactive means due to presence of treatment main effect.  ^a,b^Within an item, treatment means differ (*P* < 0.10). | | | | | | | | |  |

| Supplementary Table 4. Effects of Cu, Zn, and Mn source and inclusion during late gestation on colostrum and milk nutrient components | | | | | | | | |  |
| --- | --- | --- | --- | --- | --- | --- | --- | --- | --- |
|  | Treatment^1^ | | | |  | *P*-values | | | |
| Variable | CON | ITM | MMHAC | RR | SEM | Trt | Day | Trt × Day | |
| Colostrum^2^ |  |  |  |  |  |  |  |  | |
| Nutrient concentration |  |  |  |  |  |  |  |  | |
| Lactose, g/dL | 2.48^ab^ | 2.24^b^ | 2.88^a^ | 2.12^b^ | 0.24 | 0.08 |  |  | |
| Triglycerides, g/dL | 4.14^b^ | 5.23^ab^ | 4.16^b^ | 6.34^a^ | 0.83 | 0.10 |  |  | |
| Protein, g/dL | 16.7^bc^ | 19.6^ab^ | 13.5^c^ | 20.3^a^ | 1.7 | 0.02 |  |  | |
| Urea N, mg/dL | 4.47 | 4.73 | 4.21 | 4.45 | 0.33 | 0.67 |  |  | |
| Nutrient total^3^ |  |  |  |  |  |  |  |  | |
| Lactose, g | 29.2^ab^ | 20.5^b^ | 45.9^a^ | 17.3^b^ | 8.4 | 0.05 |  |  | |
| Triglycerides, g | 43.1 | 42.3 | 56.6 | 37.4 | 10.5 | 0.52 |  |  | |
| Protein, g | 160 | 151 | 161 | 128 | 25 | 0.65 |  |  | |
| Urea N, mg | 50.2 | 40.2 | 66.4 | 32.9 | 12.4 | 0.18 |  |  | |
|  |  |  |  |  |  |  |  |  | |
| Milk^4^ |  |  |  |  |  |  |  |  | |
| Nutrient concentration |  |  |  |  |  |  |  |  | |
| Lactose, g/dL |  |  |  |  |  | 0.75 | 0.20 | 0.18 | |
| d 35 | 4.43 | 4.48 | 4.44 | 4.59 | 0.06 |  |  |  | |
| d 60 | 4.44 | 4.35 | 4.52 | 4.40 | 0.09 |  |  |  | |
| Triglycerides, g/dL |  |  |  |  |  | 0.72 | <0.001 | 0.96 | |
| d 35 | 4.30 | 4.57 | 4.36 | 4.59 | 0.36 |  |  |  | |
| d 60 | 3.72 | 4.01 | 3.55 | 3.93 | 0.30 |  |  |  | |
| Protein, g/dL |  |  |  |  |  | 0.68 | 0.64 | 0.46 | |
| d 35 | 4.28 | 4.44 | 4.25 | 4.29 | 0.17 |  |  |  | |
| d 60 | 4.39 | 4.35 | 4.15 | 4.53 | 0.18 |  |  |  | |
| Urea N, mg/dL |  |  |  |  |  | 0.57 | <0.001 | 0.38 | |
| d 35 | 18.2 | 17.6 | 16.2 | 16.7 | 1.1 |  |  |  | |
| d 60 | 14.2 | 12.3 | 13.7 | 12.8 | 1.1 |  |  |  | |
| Nutrient total^5^ |  |  |  |  |  |  |  |  | |
| Lactose, g |  |  |  |  |  | 0.77 | 0.19 | 0.48 | |
| d 35 | 449 | 445 | 463 | 466 | 36 |  |  |  | |
| d 60 | 448 | 410 | 470 | 410 | 33 |  |  |  | |
| Triglycerides, g |  |  |  |  |  | 1.00 | 0.003 | 1.00 | |
| d 35 | 454 | 470 | 463 | 458 | 61 |  |  |  | |
| d 60 | 374 | 377 | 367 | 367 | 43 |  |  |  | |
| Protein, g |  |  |  |  |  | 1.00 | 0.76 | 0.72 | |
| d 35 | 416 | 437 | 442 | 431 | 35 |  |  |  | |
| d 60 | 443 | 420 | 423 | 421 | 31 |  |  |  | |
| Urea N, g |  |  |  |  |  | 0.48 | <0.001 | 0.88 | |
| d 35 | 1.93 | 1.78 | 1.73 | 1.76 | 0.19 |  |  |  | |
| d 60 | 1.46 | 1.16 | 1.30 | 1.22 | 0.13 |  |  |  | |
| ^1^Cows were individually-fed hay and supplemented with: no additional Cu, Zn, or Mn (control, CON), sulfate-based Cu, Zn, and Mn (inorganic, ITM) or methionine hydroxy analogue chelates of Cu, Zn, and Mn (MMHAC) to meet 133% of recommendations, or a combination of inorganic and chelated Cu, Zn, and Mn (reduce and replace, RR) to meet 100% of recommendations from 91.2 ± 6.2 d pre-calving until 11.0 ± 3.2 d post-calving.  ^2^CON *n* = 10, ITM *n* = 9, MMHAC *n* = 7, RR *n* = 9.  ^3^Total nutrients from a single rear quarter, pre-suckling.  ^4^At d 35: CON *n* = 11, ITM *n* = 11, MMHAC *n* = 9, RR *n* = 11. At d 60: CON *n* = 11, ITM *n* = 10, MMHAC *n* = 9, RR *n* = 10.  ^5^Total nutrients were calculated as concentration multiplied by 24-h milk yield.  ^a,b,c^Within an item, treatment means differ (*P* < 0.10). | | | | | | | | |  |

| Supplementary Table 5. Effects of Cu, Zn, and Mn source and inclusion during late gestation on pre-weaning calf circulating glucose, urea N, and non-esterified fatty acids (NEFA)^1^ | | | | | | | | |  |
| --- | --- | --- | --- | --- | --- | --- | --- | --- | --- |
|  | Treatment^2^ | | | |  | *P*-value | | | |
| Variable | CON | ITM | MMHAC | RR | SEM | Trt | Day | Trt × Day | |
| Plasma glucose, mg/dL |  |  |  |  |  | 0.70 | <0.001 | 0.74 | |
| d 35 | 112 | 116 | 117 | 113 | 3 |  |  |  | |
| d 60 | 105 | 105 | 104 | 102 | 3 |  |  |  | |
| d 125 | 86 | 89 | 83 | 86 | 3 |  |  |  | |
| Weaning | 82 | 83 | 85 | 83 | 3 |  |  |  | |
| Serum urea N, mg/dL |  |  |  |  |  | 0.74 | <0.001 | 0.59 | |
| d 35 | 8.2 | 9.1 | 7.8 | 8.4 | 0.6 |  |  |  | |
| d 60 | 9.2 | 9.4 | 8.9 | 9.2 | 0.5 |  |  |  | |
| d 125 | 11.3 | 11.5 | 11.4 | 12.1 | 0.6 |  |  |  | |
| Weaning | 10.9 | 10.1 | 10.2 | 10.4 | 0.6 |  |  |  | |
| Serum NEFA, µEq/L |  |  |  |  |  | 0.46 | <0.001 | 0.93 | |
| d 35 | 325 | 362 | 355 | 319 | 39 |  |  |  | |
| d 60 | 329 | 316 | 360 | 295 | 37 |  |  |  | |
| d 125 | 227 | 230 | 225 | 220 | 20 |  |  |  | |
| Weaning | 446 | 387 | 420 | 371 | 43 |  |  |  | |
| ^1^CON *n* = 11, ITM *n* = 11, MMHAC *n* = 9, RR *n* = 12.  ^2^Calves born to cows individually-fed hay and supplemented with: no additional Cu, Zn, or Mn (control, CON), sulfate-based Cu, Zn, and Mn (inorganic, ITM) or methionine hydroxy analogue chelates of Cu, Zn, and Mn (MMHAC) to meet 133% of recommendations, or a combination of inorganic and chelated Cu, Zn, and Mn (reduce and replace, RR) to meet 100% of recommendations from 91.2 ± 6.2 d pre-calving until 11.0 ± 3.2 d post-calving | | | | | | | | |  |
